# Supplementary material for: General practitioner visits after SARS-CoV-2 omicron compared with the delta variant in children in Norway: a prospective nationwide registry study
Source: BMJ Paediatr Open. 2022 Aug 26;6(1):e001502. doi: 10.1136/bmjpo-2022-001502 (PMC9421916; doi:10.1136/bmjpo-2022-001502)
Supplement: Supplementary data [file bmjpo-2022-001502supp001.pdf]

Supplementary online content

Arntzen et al. General practitioner visits after SARS-CoV-2 omicron compared to the delta variant in children in Norway: a prospective nationwide registry study

Supplemental Methods

Part 1: Data sources

In this study, we utilized the Norwegian preparedness registry (Beredt C19) to retrieve and link nation-wide individual-level data originating from the sources listed in Supplementary Table 3.

Table 1. Data sources in Beredt C19 used in this study and information obtained from each source.

**Name of data source**	**Information obtained**
The National Population Register	Resident of Norway November 1st 2021<br>Age<br>Sex<br>Municipality of residence<br>Country of birth<br>Parents' country of birth
The Norway Control and Payment of Health Reimbursement	All physical and electronic consultations with all general practitioners<br>Date of consultation
The Norwegian Surveillance System for Communicable Diseases	Date of sample of SARS-CoV-2 positive test<br>Classification of SARS-CoV-2 variants by targeted commercial or in-house PCR analyses for variant detection, Sanger sequencing of selected parts of the viral genome or whole genome sequencing.<br>For continuous surveillance purposes, 25% of SARS-CoV-2 positive samples or up to 100 samples per week per local laboratory is sent to a reference laboratory for whole genome sequencing. When Omicron emerged in Norway in late November 2021, the laboratories were requested to perform variant analyses locally on all positive samples. If this was not possible for capacity reasons, samples suspected to contain the Omicron variant were prioritized for variant analyses.
The National Immunization Register	Vaccination status by January 23rd, 2022<br>There is yet no recommended general vaccination for children below 12 years of age.
Information was linked at the individual level using the unique personal identification number	

(encrypted version) provided to every Norwegian resident at birth or upon immigration.

Supplemental Table 2: Event study estimates from the sensitivity analysis of the effect of relative week according to week Delta and Omicron variant on GP use from the 15th of November 2021 until 6th of February 2022.

Relative Week	OR	Std. Err.	P-value	Lower CI	Upper CI
**Omicron**					
Week -5	1.01	0.11	0.90	0.82	1.26
Week -4	1.10	0.08	0.23	0.94	1.28
Week -3	0.95	0.08	0.54	0.81	1.12
Week -2	0.95	0.07	0.48	0.81	1.10
Week -1	1			1	1
Week 0	6.37	0.62	0.00	5.26	7.71
Week 1	5.18	0.77	0.00	3.86	6.94
Week 2	1.42	0.10	0.00	1.24	1.62
Week 3	1.47	0.11	0.00	1.27	1.70
Week 4	1.32	0.13	0.01	1.08	1.60
**Delta**					
Week -5	0.93	0.09	0.45	0.77	1.12
Week -4	1.02	0.08	0.79	0.88	1.18
Week -3	1.00	0.06	0.98	0.90	1.12
Week -2	0.92	0.05	0.11	0.84	1.02
Week -1	1			1	1
Week 0	8.19	0.54	0.00	7.20	9.32
Week 1	7.26	0.93	0.00	5.64	9.34
Week 2	1.35	0.08	0.00	1.20	1.52
Week 3	1.27	0.07	0.00	1.15	1.41
Week 4	1.33	0.07	0.00	1.20	1.48
Note: Regression results from the sensitivity analysis using a Logit model. Number of person weeks = 6,645,376. We included controls for year of birth, calendar week, region of residence, country of birth, sex and parents' country of birth. Standard errors are clustered at the municipality level.					

Supplemental Table 3A: Event study estimates from the sensitivity analysis of the effect of relative week according to week Delta and Omicron variant on GP use in children aged 0-1 years

Age 0-1						
	Odds	Ratio	Std. Err.	z	[95% Conf. Interval]	
**Omicron**						
Week -5	1.59	0.32	2.30	0.02	1.07	2.37

Week -4	1.31	0.18	1.98	0.05	1.00	1.72
Week -3	1.02	0.15	0.12	0.91	0.76	1.37
Week -2	1.11	0.16	0.72	0.47	0.83	1.49
Week -1	1.00					
Week 0	5.61	0.64	15.13	0.00	4.48	7.01
Week 1	4.24	0.55	11.21	0.00	3.30	5.46
Week 2	1.36	0.28	1.49	0.14	0.91	2.03
Week 3	0.99	0.24	-0.04	0.97	0.62	1.59
Week 4	1.51	1.11	0.56	0.57	0.36	6.36
**Delta**						
Week -5	0.37	0.37	-1.00	0.32	0.05	2.60
Week -4	1.07	0.28	0.24	0.81	0.63	1.80
Week -3	1.36	0.29	1.42	0.16	0.89	2.07
Week -2	1.27	0.21	1.46	0.14	0.92	1.76
Week -1	1.00					
Week 0	5.75	0.52	19.39	0.00	4.82	6.87
Week 1	4.47	0.96	7.01	0.00	2.94	6.80
Week 2	1.17	0.18	1.02	0.31	0.86	1.60
Week 3	1.27	0.17	1.79	0.07	0.98	1.66
Week 4	1.78	0.24	4.22	0.00	1.36	2.33

Supplemental Table 3B: Event study estimates from the sensitivity analysis of the effect of relative week according to week Delta and Omicron variant on GP use in children aged 2-5 years

Age 2-5						
	Odds	Ratio	Std. Err.	z	[95% Conf. Interval]	
**Omicron**						
Week -5	1.15	0.12	1.35	0.18	0.94	1.41
Week -4	1.15	0.12	1.32	0.19	0.93	1.42
Week -3	1.00	0.12	0.03	0.98	0.79	1.28

Week -2	0.97	0.15	-0.19	0.85	0.72	1.31
Week -1	1.00					
Week 0	6.04	0.70	15.40	0.00	4.80	7.59
Week 1	4.82	0.71	10.75	0.00	3.62	6.43
Week 2	1.47	0.21	2.70	0.01	1.11	1.95
Week 3	1.48	0.25	2.30	0.02	1.06	2.08
Week 4	1.08	0.57	0.15	0.88	0.38	3.04
**Delta**						
Week -5	1.62	0.44	1.77	0.08	0.95	2.75
Week -4	1.31	0.28	1.25	0.21	0.86	1.98
Week -3	1.27	0.23	1.37	0.17	0.90	1.80
Week -2	1.11	0.15	0.75	0.45	0.85	1.44
Week -1	1.00					
Week 0	6.70	0.55	22.97	0.00	5.70	7.88
Week 1	5.66	0.62	15.78	0.00	4.56	7.02
Week 2	1.69	0.16	5.55	0.00	1.41	2.04
Week 3	1.25	0.12	2.38	0.02	1.04	1.50
Week 4	1.48	0.15	3.89	0.00	1.21	1.80

Supplemental Table 3C: Event study estimates from the sensitivity analysis of the effect of relative week according to week Delta and Omicron variant on GP use in children aged 6-10 years

Age 6-10						
	Odds	Ratio	Std. Err.	z	[95% Conf. Interval]	
**Omicron**						
Week -5	0.74	0.09	-2.45	0.01	0.58	0.94
Week -4	0.92	0.08	-0.97	0.33	0.79	1.08
Week -3	0.96	0.12	-0.37	0.71	0.75	1.21
Week -2	0.79	0.10	-1.77	0.08	0.61	1.03
Week -1	1.00					

Week 0	7.10	0.82	17.00	0.00	5.66	8.89
Week 1	6.07	0.83	13.12	0.00	4.63	7.94
Week 2	1.53	0.15	4.33	0.00	1.26	1.85
Week 3	1.28	0.18	1.74	0.08	0.97	1.69
Week 4	0.51	0.18	-1.92	0.05	0.25	1.01
**Delta**						
Week -5	0.72	0.25	-0.93	0.35	0.37	1.43
Week -4	1.15	0.20	0.79	0.43	0.82	1.60
Week -3	1.14	0.17	0.89	0.38	0.85	1.53
Week -2	0.85	0.10	-1.45	0.15	0.68	1.06
Week -1	1.00					
Week 0	9.39	0.62	33.97	0.00	8.25	10.69
Week 1	8.36	1.16	15.29	0.00	6.36	10.97
Week 2	1.35	0.09	4.26	0.00	1.17	1.54
Week 3	1.25	0.09	2.98	0.00	1.08	1.45
Week 4	1.15	0.10	1.59	0.11	0.97	1.38

Supplemental table 4: Event study estimates of the effect of relative week according to week delta and omicron variant on GP use from the 29th of November 2021 until 23rd of January 2022, adjusted for municipality-specific time trends.

Supplemetal Table 4: Event study estimates from the sensitivity analysis of the effect of relative week according to week Delta and Omicron variant on GP use, adjusted for continuous time-by-municipality fixed effects

justert for kommune-tids-trender							
		Odds	Std. Err.	z	p	[95% Conf. Interval]	
	Omicron						
	-5	1.02	0.11	0.24	0.81	0.84	1.25

	-4	1.07	0.08	0.96	0.34	0.93	1.23
	-3	0.97	0.08	-0.30	0.77	0.83	1.15
	-2	0.91	0.08	-1.12	0.27	0.77	1.08
	-1	0.00					
	0	6.65	0.69	18.30	0.00	5.43	8.14
	1	5.48	0.72	12.92	0.00	4.23	7.09
	2	1.50	0.12	5.10	0.00	1.29	1.76
	3	1.31	0.14	2.49	0.01	1.06	1.63
	4	0.85	0.29	-0.47	0.64	0.43	1.67
	Delta						
	-5	0.98	0.24	-0.07	0.94	0.61	1.59
	-4	1.19	0.18	1.13	0.26	0.88	1.61
	-3	1.21	0.11	2.07	0.04	1.01	1.44
	-2	0.97	0.07	-0.45	0.65	0.83	1.12
	-1	0.00					
	0	8.21	0.53	32.80	0.00	7.24	9.31
	1	7.10	0.93	14.96	0.00	5.49	9.18
	2	1.39	0.08	5.65	0.00	1.24	1.56
	3	1.26	0.08	3.67	0.00	1.11	1.42
	4	1.34	0.08	4.87	0.00	1.19	1.50
