# Supplementary material for: General practitioner visits after SARS-CoV-2 omicron compared with the delta variant in children in Norway: a prospective nationwide registry study
Source: BMJ Paediatr Open. 2022 Aug 26;6(1):e001502. doi: 10.1136/bmjpo-2022-001502 (PMC9421916; doi:10.1136/bmjpo-2022-001502)
Supplement: Author's manuscript [file bmjpo-2022-001502.draft_revisions.pdf]

BMJ Paediatrics Open

BMJ Paediatrics Open is committed to open peer review. As part of this commitment we make the peer review history of every article we publish publicly available.

When an article is published we post the peer reviewers' comments and the authors' responses online. We also post the versions of the paper that were used during peer review. These are the versions that the peer review comments apply to.

The versions of the paper that follow are the versions that were submitted during the peer review process. They are not the versions of record or the final published versions. They should not be cited or distributed as the published version of this manuscript.

BMJ Paediatrics Open is an open access journal and the full, final, typeset and author-corrected version of record of the manuscript is available on our site with no access controls, subscription charges or pay-per-view fees (http://bmjpaedsopen.bmj.com).

If you have any questions on BMJ Paediatrics Open's open peer review process please email info.bmjpo@bmj.com

BMJ Paediatrics Open

General practitioner visits after SARS-CoV-2 Omicron compared to the Delta variant in children: a prospective nationwide registry study

Journal:	*BMJ Paediatrics Open*
Manuscript ID	bmjpo-2022-001502
Article Type:	Original research letter
Date Submitted by the Author:	05-Apr-2022

Complete List of Authors:	Arntzen, Sigurd Storehaug; Norwegian Institute of Public Health<br>Gjefsen, Hege Marie; Norwegian Institute of Public Health<br>Telle, Kjetil Elias; Norwegian Institute of Public Health<br>Magnusson, Karin; Norwegian Institute of Public Health<br>Stordal, Ketil; Norwegian Institute of Public Health<br>Håberg, Siri; Norwegian Institute of Public Health, Institute Management and Staff<br>Kinge, Jonas; Norwegian Institute of Public Health

Keywords:	COVID-19

SCHOLARONE™
Manuscripts

I, the Submitting Author has the right to grant and does grant on behalf of all authors of the Work (as defined in the below author licence), an exclusive licence and/or a non-exclusive licence for contributions from authors who are: i) UK Crown employees; ii) where BMJ has agreed a CC-BY licence shall apply, and/or iii) in accordance with the terms applicable for US Federal Government officers or employees acting as part of their official duties; on a worldwide, perpetual, irrevocable, royalty-free basis to BMJ Publishing Group Ltd ("BMJ") its licensees and where the relevant Journal is co-owned by BMJ to the co-owners of the Journal, to publish the Work in this journal and any other BMJ products and to exploit all rights, as set out in our licence.

The Submitting Author accepts and understands that any supply made under these terms is made by BMJ to the Submitting Author unless you are acting as an employee on behalf of your employer or a postgraduate student of an affiliated institution which is paying any applicable article publishing charge ("APC") for Open Access articles. Where the Submitting Author wishes to make the Work available on an Open Access basis (and intends to pay the relevant APC), the terms of reuse of such Open Access shall be governed by a Creative Commons licence – details of these licences and which Creative Commons licence will apply to this Work are set out in our licence referred to above.

Other than as permitted in any relevant BMJ Author's Self Archiving Policies, I confirm this Work has not been accepted for publication elsewhere, is not being considered for publication elsewhere and does not duplicate material already published. I confirm all authors consent to publication of this Work and authorise the granting of this licence.

Confidential: For Review Only

General practitioner visits after SARS-CoV-2 Omicron compared to the Delta variant in children: a prospective nationwide registry study

Sigurd Storehaug Arntzen, MPhil[1], Hege Marie Gjefsen, PhD[1], Kjetil Elias Telle, PhD[1], Karin Magnusson, PhD[1], Ketil Størdal, MD PhD[2], Siri Håberg, MD PhD[3], Jonas Minet Kinge, PhD[134]

[1] Cluster for Health Services Research, Norwegian Institute of Public Health
[2] Division of Paediatric and Adolescent Medicine, University of Oslo
[3] Centre for Fertility and Health, Norwegian Institute of Public Health
[4] Department of Health Management and Health Economics, University of Oslo

Address correspondence to: Jonas Minet Kinge, Norwegian Institute of Public Health, Postboks 222, Skøyen, N-0213 Oslo
E-mail: jonas.minet.kinge@fhi.no.

Conflict of Interest Disclosures (includes financial disclosures): The authors have no example conflicts of interest to disclose.

Funding/Support: Funding: The study was internally funded by the Norwegian Institute of Public Health and externally by the Research Council of Norway (project no 262700). The funding sources had no influence over the design and conduct of the study; collection, management, analysis, and interpretation of the data; preparation, review, or approval of the manuscript; or decision to submit the manuscript for publication.

Word Count: 508

Confidential: For Review Only

SARS-CoV-2 in children leads to milder symptoms than in adults.[1] However, recent reports discuss whether the omicron variant might cause more severe symptoms than the delta variant in children.[2] In Norway, general practitioners (GP) serve as the first line health service and are free for children aged below 16. Based on national recommendations, healthy children below age 12 are mostly unvaccinated.[3] PCR tests are freely available and a proportion of these are screened for variants.

The aim of this analysis was to compare GP contacts among children in the five weeks after being infected with omicron or delta variants.

Using data from the Emergency Preparedness Register (Beredt C19), we followed Norwegian residents aged 0-10 years from the 29th of November 2021 until 23rd of January 2022 (Supplemental Table 1). The outcome variable was defined as at least one physical- or e-consultation with a GP in a week. Children who tested positive but whose tests were not sequenced, and children who had been vaccinated, were excluded from the analysis. Multivariate logistic regression was used to estimate adjusted odds ratios (aOR) with 95% confidence intervals (CI) for GP consultations. Exploiting the longitudinal nature of the data, we used an event-study design, controlling for calendar week of consultation, municipality fixed effects and sociodemographic controls. The temporal aORs of being infected by omicron and delta were estimated from 5 weeks before to 4 weeks after the week 0 of infection (Supplemental Methods part 2). The event-study allows for testing whether infected children followed the same patterns for GP visits as the noninfected.[4] The reference category was 1 week prior to infection and noninfected individuals were included as controls. We also varied the time period to explore the robustness of our findings.

Confidential: For Review Only

In total, 661,587 children, aged 0–10 years, were included in the study. After excluding 47,683 children with positive test during the study period that were not sequenced and 474 children who were vaccinated, the primary study population consisted of 613,448 children comprising 4,907,584 person-weeks (Table). The delta variant was the dominant strain at the beginning and omicron was the dominant strain at the end of the study period (Supplemental Figure 1).

GP consultations in the weeks before infection were similar for the children with omicron and delta in the adjusted model (See the Figure and Supplemental Tables 2, 3 & 4). The aORs in the first and second week after positive tests were higher for delta than for omicron. Higher GP utilization was also found for children 4 and 5 weeks after testing positive for delta but returned to pre-infection levels in week 5 for omicron cases. Results from robustness control were similar (Supplemental Table 4).

Strengths of this study includes the use of data covering all Norwegian children and that both delta and omicron was circulating during our study period. A limitation of the study is that asymptomatic children may have been less likely to have had a test. If for example, asymptomatic SARS-CoV-2 was more common for the omicron virus, we might have underestimated the difference between the two strains.

Patient and public involvement

No patients were involved in setting the research question or the outcome measures, nor were they involved in developing plans for recruitment, design, or implementation of the study. No patients were asked to advise on interpretation or writing up of results. There are no plans to disseminate the results of the research to study participants or the relevant patient community.

Confidential: For Review Only

Funding

The study was internally funded by the Norwegian Institute of Public Health and externally by the Research Council of Norway (project no 262700).

Acknowledgments

The establishment of an emergency preparedness register forms part of the legally mandated responsibilities of the Norwegian Institute of Public Health during epidemics. The Ethics Committee of South-East Norway confirmed (4 June 2020, #153204) that external ethical board review was not required.

References:

1. Lee B, Raszka WV, Jr. COVID-19 in Children: Looking Forward, Not Back. Pediatrics 2021;147(1). DOI: 10.1542/peds.2020-029736.
2. Kozlov M. Does Omicron hit kids harder? Scientists are trying to find out. Nature Web Site.
3. Coronavirus vaccine - information for the public. The Norwegian Institute of Public Health (NIPH). 28.02.2022 (https://www.fhi.no/en/id/vaccines/coronavirus-immunisation-programme/coronavirus-vaccine/#vaccination-of-children-and-adolescents).
4. Dimick JB, Ryan AM. Methods for Evaluating Changes in Health Care Policy: The Difference-in-Differences Approach. JAMA 2014;312(22):2401-2402. DOI: 10.1001/jama.2014.16153.

Confidential: For Review Only

Table: Summary statistics of the estimation sample

	Omicron	Delta	Rest
**N**	7,046	14,369	613,448
**Person-weeks**	56,368	114,952	4,907,584
**Age, mean (SD)**	6.1 (3.1)	6.9(2.8)	5.2 (3.2)
**Born Foreign Country, (%)**	7.2	6.3	5.5
**Parents foreign Born Country, (%)**	53.7	43.0	38.8
**Boys, (%)**	51.3	51.4	51.3

Notes: Standard deviation in brackets

Figure: Event-study analysis of pre- and post-SARS-COV-2 trends in odds ratios for GP utilization by variant

[Figure]

Note: Estimates from logistic regression analysis on weekly data are reported. Adjusted odds ratios were estimated for indicator variables for relative week to omicron and delta and the omitted category defined as one week prior to infection, i.e week -1. Odds ratios were adjusted for age by including age indicators for 1 year age groups, sex, indicator for birth country and parental birth country, and municipality of residence. Standard errors are clustered at municipality level.

Confidential: For Review Only

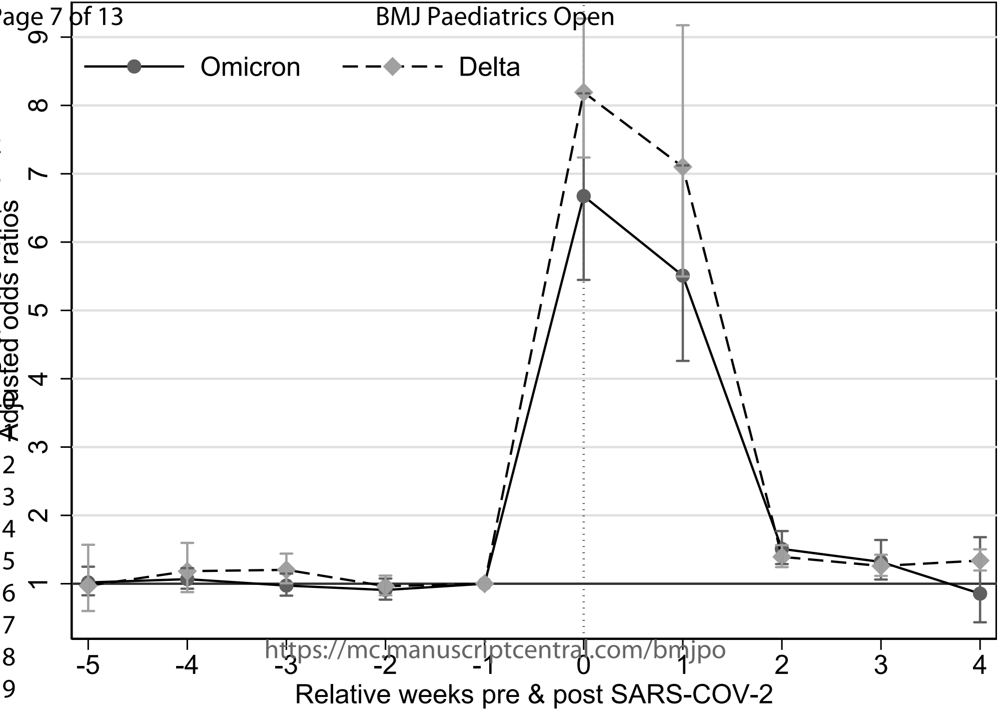

https://mc.manuscriptcentral.com/bmjpo

Supplementary online content

Arntzen et al. General practitioner visits after SARS-CoV-2 Omicron compared to the Delta variant in children: a prospective nationwide registry study

Confidential: For Review Only

Supplemental Methods

Part 1: Data sources

In this study, we utilized the Norwegian preparedness registry (Beredt C19) to retrieve and link nation-wide individual-level data originating from the sources listed in Supplementary Table 3.

Table 1. Data sources in Beredt C19 used in this study and information obtained from each source.

**Name of data source**	**Information obtained**
The National Population Register	Resident of Norway November 1st 2021<br>Age<br>Sex<br>Municipality of residence<br>Country of birth<br>Parents' country of birth
The Norway Control and Payment of Health Reimbursement	All physical and electronic consultations with all general practitioners<br>Date of consultation
The Norwegian Surveillance System for Communicable Diseases	Date of sample of SARS-CoV-2 positive test<br><br>Classification of SARS-CoV-2 variants by targeted commercial or in-house PCR analyses for variant detection, Sanger sequencing of selected parts of the viral genome or whole genome sequencing.<br><br>For continuous surveillance purposes, 25% of SARS-CoV-2 positive samples or up to 100 samples per week per local laboratory is sent to a reference laboratory for whole genome sequencing. When Omicron emerged in Norway in late November 2021, the laboratories were requested to perform variant analyses locally on all positive samples. If this was not possible for capacity reasons, samples suspected to contain the Omicron variant were prioritized for variant analyses.
The National Immunization Register	Vaccination status by January 23rd, 2022<br><br>There is yet no recommended general vaccination for children below 12 years of age.

Confidential: For Review Only

Information was linked at the individual level using the unique personal identification number (encrypted version) provided to every Norwegian resident at birth or upon immigration.

Part 2: Statistical methods

Our empirical strategy is:

$$y_{i,w} = \theta_w + \theta_i + D_{\delta,i} \sum_{k=-5,\ k\neq-1}^{k=5} \delta_{k(i,w)}\alpha_k + D_{\sigma,i} \sum_{k=-5,\ k\neq-1}^{k=5} \sigma_{k(i,w)}\beta_k + \epsilon_{i,w}$$

Here $y_{i,w}$ is the outcome in week *w* for individual *i*. $D_{\delta,i}$ is a dummy indicating whether individual *i* was infected with the Delta virus in our study period, while $D_{\sigma,i}$ is a dummy indicating whether individual *i* was infected with the omicron virus. δ_k is a set of time variables, indicating that there are *k* weeks relative to the week in which the individual got infected with the delta virus. Similarly, $\sigma_k$indicates that there are *k* weeks relative to the week in which the individual got infected with the omicron virus. θ_w are event-specific fixed effects for calendar week *w* , θ_i denotes background characteristics for individual *i* including gender, age dummies, parental country background and the child's country background, as well as municipality fixed effects. $\epsilon_{i,w}$ is the usual standard error clustered at the municipality level.

Our primary parameters of interests are the α_k and β_k attached to the event time dummies. These will capture the changes in our outcome variable among the children infected with delta and omicron relative to the comparison group. The coefficients for k < 0 indicate the pre-trends prior to infection time, while k > 0 describe how the outcome changes after getting infected with either delta or omicron virus.

Confidential: For Review Only

Supplemental Results

Supplemental Figure 1: The development of delta and omicron cases in the estimated sample, week 48, 2021 – week 3, 2022. Share of sequenced samples with confirmed delta, omicron, and other results.

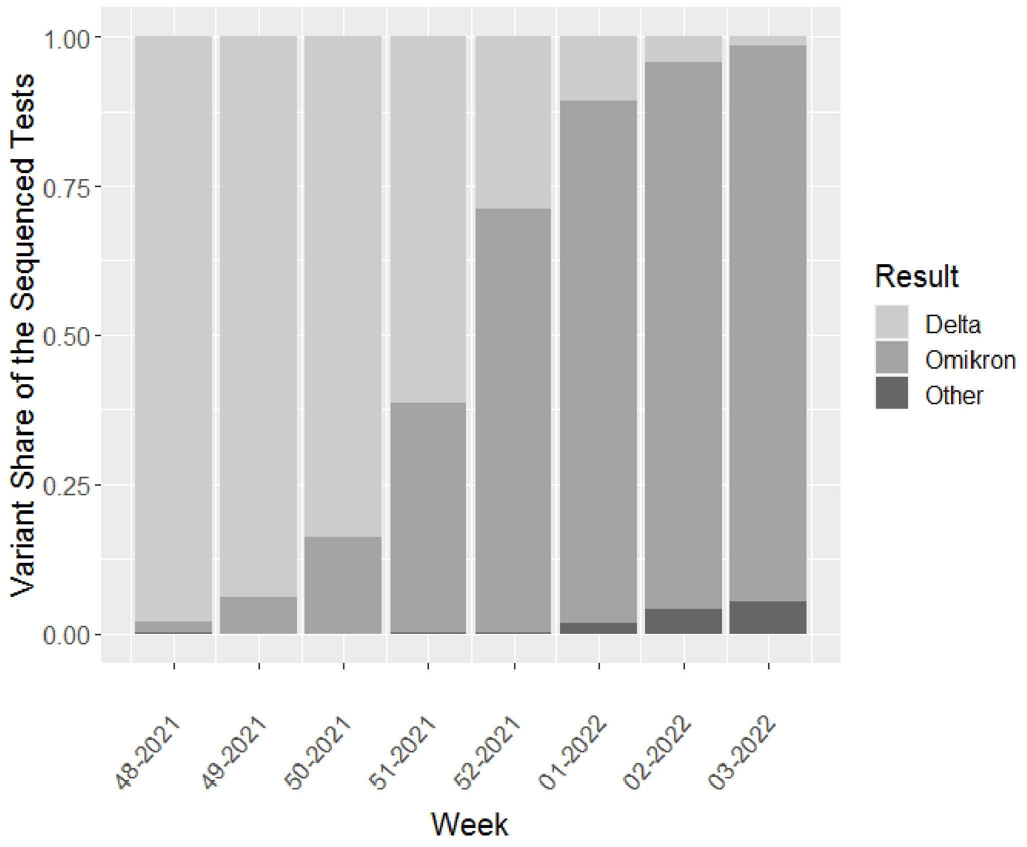

For peer review Only

Supplemental Table 2: Descriptive statistics of the estimated sample

	Delta			Omicron		
Relative week	Person-weeks with GP Appointment	Persons infected	Fraction with GP appointment	Person-weeks with GP appointment	Persons infected	Share with GP appointment
-5	19	692	0.03	145	4,975	0.03
-4	55	1,622	0.03	176	6,126	0.03
-3	113	3,452	0.03	147	6,600	0.02
-2	187	7,445	0.03	120	6,902	0.02
-1	259	11,636	0.02	125	7,024	0.02
0	2,285	14,369	0.16	846	7,046	0.12
1	1,795	14,351	0.13	674	6,263	0.11
2	335	14,177	0.02	136	4,133	0.03
3	282	13,677	0.02	60	2,071	0.03
4	304	12,747	0.02	17	920	0.02

Confidential: For Review Only

Supplemental Table 3: Event study estimates of the effect of relative week according to week Delta and Omicron variant on GP use from the 29th of November 2021 until 23rd of January 2022.

Relative Week	OR	Std. Err.	P-value	Lower CI	Upper CI
**Omicron**					
Week -5	1.20	0.10	0.03	1.02	1.42
Week -4	1.02	0.11	0.86	0.83	1.25
Week -3	1.07	0.08	0.37	0.93	1.23
Week -2	0.97	0.08	0.75	0.83	1.15
Week -1	0.91	0.08	0.27	0.77	1.08
Week 0	6.67	0.69	0.00	5.45	8.18
Week 1	5.51	0.72	0.00	4.26	7.12
Week 2	1.51	0.12	0.00	1.29	1.77
Week 3	1.32	0.15	0.01	1.06	1.64
Week 4	0.86	0.29	0.65	0.44	1.68
**Delta**					
Week -5	0.58	0.37	0.39	0.16	2.04
Week -4	0.97	0.24	0.90	0.60	1.57
Week -3	1.18	0.18	0.27	0.88	1.60
Week -2	1.20	0.11	0.04	1.01	1.44
Week -1	0.96	0.07	0.63	0.83	1.12
Week 0	8.19	0.52	0.00	7.24	9.27
Week 1	7.10	0.93	0.00	5.50	9.17
Week 2	1.39	0.08	0.00	1.24	1.56
Week 3	1.26	0.08	0.00	1.11	1.42
Week 4	1.34	0.08	0.00	1.19	1.50

Note: Regression results from the main specification using a Logit model. Number of person weeks = 4,907,584. We included controls for year of birth, calendar week, region of residence, country of birth, sex and parents' country of birth. Standard errors are clustered at the municipality level.

Confidential: For Review Only

Supplemental Table 4: Event study estimates from the sensitivity analysis of the effect of relative week according to week Delta and Omicron variant on GP use from the 15th of November 2021 until 6th of February 2022.

Relative Week	OR	Std. Err.	P-value	Lower CI	Upper CI
**Omicron**					
Week -5	1.21	0.06	0.00	1.09	1.33
Week -4	1.01	0.11	0.90	0.82	1.26
Week -3	1.10	0.08	0.23	0.94	1.28
Week -2	0.95	0.08	0.54	0.81	1.12
Week -1	0.95	0.07	0.48	0.81	1.10
Week 0	6.37	0.62	0.00	5.26	7.71
Week 1	5.18	0.77	0.00	3.86	6.94
Week 2	1.42	0.10	0.00	1.24	1.62
Week 3	1.47	0.11	0.00	1.27	1.70
Week 4	1.32	0.13	0.01	1.08	1.60
**Delta**					
Week -5	0.95	0.17	0.76	0.66	1.35
Week -4	0.93	0.09	0.45	0.77	1.12
Week -3	1.02	0.08	0.79	0.88	1.18
Week -2	1.00	0.06	0.98	0.90	1.12
Week -1	0.92	0.05	0.11	0.84	1.02
Week 0	8.19	0.54	0.00	7.20	9.32
Week 1	7.26	0.93	0.00	5.64	9.34
Week 2	1.35	0.08	0.00	1.20	1.52
Week 3	1.27	0.07	0.00	1.15	1.41
Week 4	1.33	0.07	0.00	1.20	1.48

Note: Regression results from the sensitivity analysis using a Logit model. Number of person weeks = 6,645,376. We included controls for year of birth, calendar week, region of residence, country of birth, sex and parents' country of birth. Standard errors are clustered at the municipality level.

Confidential: For Review Only

BMJ Paediatrics Open

General practitioner visits after SARS-CoV-2 omicron compared to the delta variant in children: a prospective nationwide registry study

Journal:	*BMJ Paediatrics Open*
Manuscript ID	bmjpo-2022-001502.R1
Article Type:	Original research
Date Submitted by the Author:	03-May-2022
Complete List of Authors:	Arntzen, Sigurd Storehaug; Norwegian Institute of Public Health<br>Gjefsen, Hege Marie; Norwegian Institute of Public Health<br>Telle, Kjetil Elias; Norwegian Institute of Public Health<br>Magnusson, Karin; Norwegian Institute of Public Health<br>Stordal, Ketil; Norwegian Institute of Public Health<br>Håberg, Siri; Norwegian Institute of Public Health, Institute Management and Staff<br>Kinge, Jonas; Norwegian Institute of Public Health
Keywords:	COVID-19

SCHOLARONE™
Manuscripts

I, the Submitting Author has the right to grant and does grant on behalf of all authors of the Work (as defined in the below author licence), an exclusive licence and/or a non-exclusive licence for contributions from authors who are: i) UK Crown employees; ii) where BMJ has agreed a CC-BY licence shall apply, and/or iii) in accordance with the terms applicable for US Federal Government officers or employees acting as part of their official duties; on a worldwide, perpetual, irrevocable, royalty-free basis to BMJ Publishing Group Ltd ("BMJ") its licensees and where the relevant Journal is co-owned by BMJ to the co-owners of the Journal, to publish the Work in this journal and any other BMJ products and to exploit all rights, as set out in our licence.

The Submitting Author accepts and understands that any supply made under these terms is made by BMJ to the Submitting Author unless you are acting as an employee on behalf of your employer or a postgraduate student of an affiliated institution which is paying any applicable article publishing charge ("APC") for Open Access articles. Where the Submitting Author wishes to make the Work available on an Open Access basis (and intends to pay the relevant APC), the terms of reuse of such Open Access shall be governed by a Creative Commons licence – details of these licences and which Creative Commons licence will apply to this Work are set out in our licence referred to above.

Other than as permitted in any relevant BMJ Author's Self Archiving Policies, I confirm this Work has not been accepted for publication elsewhere, is not being considered for publication elsewhere and does not duplicate material already published. I confirm all authors consent to publication of this Work and authorise the granting of this licence.

Confidential: For Review Only

General practitioner visits after SARS-CoV-2 omicron compared to the delta variant in children: a prospective nationwide registry study

Sigurd Storehaug Arntzen, MPhil[1], Hege Marie Gjefsen, PhD[1], Kjetil Elias Telle, PhD[1], Karin Magnusson, PhD[1], Ketil Størdal, MD PhD[2], Siri Håberg, MD PhD[3], Jonas Minet Kinge, PhD[134]

[1] Cluster for Health Services Research, Norwegian Institute of Public Health
[2] Division of Paediatric and Adolescent Medicine, University of Oslo
[3] Centre for Fertility and Health, Norwegian Institute of Public Health
[4] Department of Health Management and Health Economics, University of Oslo

Address correspondence to: Jonas Minet Kinge, Norwegian Institute of Public Health, Postboks 222, Skøyen, N-0213 Oslo
E-mail: jonas.minet.kinge@fhi.no.

Conflict of Interest Disclosures (includes financial disclosures): The authors have no example conflicts of interest to disclose.

Funding/Support: Funding: The study was internally funded by the Norwegian Institute of Public Health and externally by the Research Council of Norway (project no 262700). The funding sources had no influence over the design and conduct of the study; collection, management, analysis, and interpretation of the data; preparation, review, or approval of the manuscript; or decision to submit the manuscript for publication.

Confidential: For Review Only

What is already known on this topic

SARS-CoV-2 in children is known to lead to an immediate increase in primary care utilization for 1-2 weeks after a positive test, before quickly falling back to pre-infection utilization levels. The difference in primary care utilization following infection with the delta and omicron virus variants is unknown.

What this study adds

A sudden increase in primary care use was observed in children who tested positive for SARS-CoV-2 in the two weeks after the test. After the first two weeks, the primary care utilization returned to pre-infection levels.

The increase in primary care utilization was higher for children infected with the delta than the omicron variant. Compared to the delta variant, the omicron variant is likely to result in less pressure on primary care services for infected children.

Confidential: For Review Only

Abstract:

Background: SARS-CoV-2 infection in children is followed by an immediate increase in primary care utilization. The difference in utilization following infection with the delta and omicron virus variants is unknown.

Objectives: To study whether general practitioner (GP) contacts are different in children infected with the omicron vs delta variant, for up to five weeks after the infection.

Setting: Primary care

Participants: All residents in Norway aged 0-10. After excluding 47,683 children with a positive test where the virus variant was not identified as delta or omicron and 474 children who were vaccinated, the primary study population consisted of 613,448 children.

Main outcome measures: GP visit

Methods: We estimated the difference in the weekly share visiting the GP after being infected with the delta or omicron variant to those in the study population who were either not tested or who tested negative, using an event-study design, controlling for calendar week of consultation, municipality fixed effects and sociodemographic factors in multivariate logistic regressions.

Results: Compared with pre-infection, increased GP utilization was found for children 1 and 2 weeks after testing positive for the omicron variant, with an odds ratio (OR) of 6.7 (Std.err: 0.69) in the first week and 5.5 (0.72) in the second. This increase was more pronounced for children with the delta variant, with an OR of 8.2 (0.52) in the first week and 7.1 (0.93) in the second. After 2 weeks, the GP utilization returned to pre-infection levels.

Conclusion: The omicron variant is likely to result in less pressure on primary health care services for children, compared with the delta variant.

Confidential: For Review Only

Confidential: For Review Only

Introduction

SARS-CoV-2 for children is often referred to as leading to milder symptoms than in adults and recent studies found no increase in specialists care in children following infection.[1 2] However, recent reports discuss whether the omicron variant might cause more severe symptoms than the delta variant in children.[3 4]

Survey data can be used to determine patterns of health care need following infection, however, reporting and response bias may affect the accuracy of the estimates. Except for a few studies using registry data from a period dominated by delta and other earlier variants, little is known about the impact of SARS-CoV-2 on post-covid health care utilization in children[2]. Furthermore, we also do not know whether healthcare use among children and adolescents increases after initial omicron infection and whether this increase, if any, is comparable to the increase in utilization after infection with the delta variant. Such knowledge could be used to upscale or downscale the health care services.

Because the omicron variant has been found to cause less severe symptoms than the delta variant in adults, we hypotezized that a comparable pattern would be found for children. The aim of this analysis was to compare general practitioner (GP) contacts among children in the five weeks after being infected with the omicron or delta variants.

Methods

Data used for this project was from the Emergency Preparedness Register (Beredt C19). The establishment of an emergency preparedness register forms part of the legally mandated responsibilities of the Norwegian Institute of Public Health during epidemics. The Ethics Committee of South-East Norway confirmed (4 June 2020, #153204) that external ethical board review was not required.

Data Sources

To estimate the share of children aged 0-10 years old visiting the GP after being infected with delta or omicron variant we used population wide longitudinal registry data from Norway. BeredtC19 is an emergency preparedness register that aims to rapidly provide ongoing overview and knowledge of the prevalence, causal relationships, and consequences of the COVID-19 epidemic in Norway. It includes information from various data sources that are updated daily, including the Norwegian surveillance system for communicable diseases (all testing and screening for SARS-CoV-2), the National Population Register (age, sex, country of birth, the National Immunization Register (vaccination status), and the Norway Control and Payment of Health Reimbursement (all physical and electronic consultations with all general practitioners). A more in-depth summary of the data sources used for our analysis are available in the Appendix Supplementary Table 1.

Study Population

We followed all Norwegian residents aged 0-10 years from the 29th of November 2021 until 23rd of January 2022. Figure 1 shows the share of the sequenced PCR tests that were delta or omicron variant from the 29th of November until the 23rd of January. Children who tested positive but whose tests were not sequenced, and children who had been vaccinated, were excluded from the analysis.

The categorical outcome variable for GP contact was set to 1 if the individual had at least one physical- or e-consultation with a GP in a week, and 0 otherwise. In Norway, consultations with the GP are free for children aged below 16. The GPs serve as the first line in the health

Confidential: For Review Only

care services, prescribing medicines and performing simple procedures, and referring patients to further care when necessary.

Statistical design

Multivariate logistic regression was used to estimate adjusted odds ratios (aOR) with 95% confidence intervals (CI) for GP consultations. Exploiting the longitudinal nature of the data, we used an event-study design[5], controlling for calendar week of consultation, municipality fixed effects and sociodemographic characteristics. The temporal aORs of being infected by omicron and delta were estimated from 5 weeks before to 4 weeks after the week 0 of infection. We regress weeks to and from confirmed positive test on binary GP visits using:

$$y_{iw} = \theta_w + \theta_i + \sum_{k=-5,\ k\neq-1}^{k=5} \delta_{k(iw)}\alpha_k + \sum_{k=-5,\ k\neq-1}^{k=5} \sigma_{k(iw)}\beta_k + \epsilon_{iw}$$

Where y_{iw} is the outcome for individual *i* in week w. δ_k is a set of time variables, indicating that there are *k* weeks relative to the week in which the individual got infected with the delta virus. Similarly, σ_k indicates that there are *k* weeks relative to the week in which the individual got infected with the omicron virus. θ_w are event-specific fixed effects for calendar week w , θ_i denotes background characteristics for individual *i* including gender, age dummies, parental country background and the child's country background, as well as municipality fixed effects. ϵ_{iw} is the standard error clustered at the municipality level. Our primary parameters of interests are the α_k and β_k attached to the event time dummies. These will capture the changes in our outcome variable among the children infected with delta and omicron relative to the comparison group consisting of uninfected or non-tested children.

The coefficients for k < 0 indicate the pre-trends prior to infection time, while k > 0 describe how the outcome changes after getting infected with either delta or omicron virus. Hence, the

Confidential: For Review Only

event-study allows for testing whether infected children followed the same patterns for GP visits as the noninfected. The reference category was 1 week prior to infection and noninfected individuals were included as controls.

In the Online Appendix we present robustness checks of our results by varying the time period of our analysis.

Patient and public involvement

No patients were involved in setting the research question or the outcome measures, nor were they involved in developing plans for recruitment, design, or implementation of the study. No patients were asked to advise on interpretation or writing up of results. There are no plans to disseminate the results of the research to study participants or the relevant patient community.

Results

In total, 661,587 children, aged 0–10 years, were residing in Norway in the study period. After excluding 474 children who were vaccinated and 47,665 children with positive tests during the study period where the virus variant was not identified as delta or omicron, the primary study population consisted of 613,448 children comprising 4,907,584 person-weeks.

Figure 1 shows that delta was the dominant virus variant at the beginning of our study period, while omicron was the dominant variant at the end of our study period. Persons with omicron were older and more often born abroad than persons with delta, though the sex distribution was similar (Table 1).

Confidential: For Review Only

Table 2 shows descriptive statistics of the estimated sample, indicating that the fraction that visited the GP in the weeks before infection was similar for the children later infected with omicron and delta, and for those who either were not infected or not tested for SARS-CoV-2.

The event study plot showing higher GP utilization following omicron, compared with delta (Figure 2). The aORs of 8.19 (std.err: 0.52) in the first and 7.10 (0.93) in the second week after delta were higher than comparable estimates for omicron of 6.67 (0.69) and 5.51 (0.72) in week one and two respectively. Higher GP utilization was also found for children 4 weeks after testing positive for both omicron and delta. Higher utilization was also found five weeks after testing positive for delta but returned to pre-infection levels in week 5 for omicron cases (see Table 3 for details). Results from robustness control were similar (Supplemental Table 2).

Discussion

In this study of 613,448 Norwegian children aged 0-10 years, we found increased GP utilization for children 1 and 2 weeks after testing positive for the omicron variant, with similar and more pronounced increases for children with the delta variant. Our findings suggest that omicron will place less pressure on the primary care services per case. However, given the higher transmissibility of the omicron than the delta variant it can still lead to a high burden on the health care system.[6]

We could find no study for comparison of our findings, i.e. to our knowledge, the present study is the first to shed light on healthcare use following the omicron vs delta variant in children.

Confidential: For Review Only

A number of reports of the omicron variant in adults suggests less serious illness[7], i.e. another possible interpretation of our findings may be that omicron is perceived as less severe, compared with the delta variant, lowering the parents' perceived need to seek healthcare after omicron than after delta. However, a key strength of our analysis is that both delta and omicron were circulating during our study period, and we adjusted for calendar week in our regression models. Hence, it is less likely that the results are driven by general changes in the inclination to contact the GP. In addition, it includes data covering all children residing in Norway, which reduce attrition and sample selection bias.

Based on national recommendations in Norway, healthy children below age 12 are mostly unvaccinated.[8] PCR tests are freely available and a proportion of these are screened for variants of concern. Several of the most populous municipalities in Norway had implemented mass testing in the schools before the first case of omicron was detected in Norway in late November 2021. However, throughout the study period, it was the norm that all positive rapid antigen home tests had to be confirmed with a PCR-test. The increasing use of rapid antigen home tests is therefore unlikely to seriously bias our estimates.

A limitation of the study is that asymptomatic children might have been less likely to have a PCR-test. If, for example, asymptomatic SARS-CoV-2 infection was more common for the omicron than the delta variant, we might have underestimated the difference between them. Also, our findings may not be generalizable to countries without equal and free access to healthcare and PCR testing for SARS-CoV-2 for all inhabitants. Finally, for continuous surveillance purposes, 25% of SARS-CoV-2 positive samples or up to 100 samples per week per local laboratory is sent to a reference laboratory for whole genome sequencing. When omicron emerged in Norway in late November 2021, the laboratories were requested to

Confidential: For Review Only

perform variant analyses locally on all positive samples. If this was not possible for capacity reasons, samples suspected to contain the omicron variant was prioritized for variant analyses. This might have led to variation in the tests that were sequenced over time.

Conclusion

Our findings show that per positive test in children aged 0 to 10 years, the omicron variant is likely to result in less pressure on the health care system than the delta variant. However, the omicron variant is still associated with increased utilization, and it can still lead to a high burden on the health care system when the community omicron cases are high.

References:

1. Dhochak N, Singhal T, Kabra S, et al. Pathophysiology of COVID-19: why children fare better than adults? *The Indian Journal of Pediatrics* 2020;87(7):537-46.
2. Magnusson K, Skyrud KD, Suren P, et al. Healthcare use in 700 000 children and adolescents for six months after covid-19: before and after register based cohort study. *BMJ* 2022;376:e066809. doi: 10.1136/bmj-2021-066809
3. Kozlov M. Does Omicron hit kids harder? Scientists are trying to find out: Nature Web Site; 2022 [accessed 23.02. 2022.
4. Sigal A, Milo R, Jassat W. Estimating disease severity of Omicron and Delta SARS-CoV-2 infections. *Nature Reviews Immunology* 2022 doi: 10.1038/s41577-022-00720-5
5. Bailey MJ, Goodman-Bacon A. The War on Poverty's Experiment in Public Medicine: Community Health Centers and the Mortality of Older Americans. *American Economic Review* 2015;105(3):1067-104. doi: 10.1257/aer.20120070
6. Jørgensen SB, Nygård K, Kacelnik O, et al. Secondary Attack Rates for Omicron and Delta Variants of SARS-CoV-2 in Norwegian Households. *JAMA* 2022 doi: 10.1001/jama.2022.3780
7. Veneti L, Bøås H, Kristoffersen AB, et al. Reduced risk of hospitalisation among reported COVID-19 cases infected with the SARS-CoV-2 Omicron BA. 1 variant compared with the Delta variant, Norway, December 2021 to January 2022. *Eurosurveillance* 2022;27(4):2200077.
8. Coronavirus vaccine - information for the public: The Norwegian Institute of Public Health (NIPH); 2022 [updated 28.02.2022. Available from: https://www.fhi.no/en/id/vaccines/coronavirus-immunisation-programme/coronavirus-vaccine/#vaccination-of-children-and-adolescents accessed 01.03. 2022.

Confidential: For Review Only

Table 1: Summary statistics of the estimation sample

	Omicron	Delta	Rest
**N**	7,046	14,369	592,033
**Person-weeks**	56,368	114,952	4,736,264
**Age, mean (SD)**	6.1 (3.1)	6.9 (2.8)	5.1 (3.2)
**Born Foreign Country, (%)**	7.2	6.3	5.5
**Parents foreign Born Country, (%)**	53.7	43.0	38.8
**Boys, (%)**	51.3	51.4	51.3

Notes: Standard deviation in brackets

Table 2: Descriptive statistics of the estimated sample

	Delta			Omicron		
Relative week	Person-weeks with GP Appointment	Persons infected	Fraction with GP appointment	Person-weeks with GP appointment	Persons infected	Share with GP appointment
-5	19	692	0.03	145	4,975	0.03
-4	55	1,622	0.03	176	6,126	0.03
-3	113	3,452	0.03	147	6,600	0.02
-2	187	7,445	0.03	120	6,902	0.02
-1	259	11,636	0.02	125	7,024	0.02
0	2,285	14,369	0.16	846	7,046	0.12
1	1,795	14,351	0.13	674	6,263	0.11
2	335	14,177	0.02	136	4,133	0.03
3	282	13,677	0.02	60	2,071	0.03
4	304	12,747	0.02	17	920	0.02

Confidential: For Review Only

Table 3: Event study estimates of the effect of relative week according to week delta and omicron variant on GP use from the 29th of November 2021 until 23rd of January 2022.

Relative Week	OR	Std. Err.	P-value	Lower CI	Upper CI
**Omicron**					
Week -5	1.20	0.10	0.03	1.02	1.42
Week -4	1.02	0.11	0.86	0.83	1.25
Week -3	1.07	0.08	0.37	0.93	1.23
Week -2	0.97	0.08	0.75	0.83	1.15
Week -1	0.91	0.08	0.27	0.77	1.08
Week 0	6.67	0.69	0.00	5.45	8.18
Week 1	5.51	0.72	0.00	4.26	7.12
Week 2	1.51	0.12	0.00	1.29	1.77
Week 3	1.32	0.15	0.01	1.06	1.64
Week 4	0.86	0.29	0.65	0.44	1.68
**Delta**					
Week -5	0.58	0.37	0.39	0.16	2.04
Week -4	0.97	0.24	0.90	0.60	1.57
Week -3	1.18	0.18	0.27	0.88	1.60
Week -2	1.20	0.11	0.04	1.01	1.44
Week -1	0.96	0.07	0.63	0.83	1.12
Week 0	8.19	0.52	0.00	7.24	9.27
Week 1	7.10	0.93	0.00	5.50	9.17
Week 2	1.39	0.08	0.00	1.24	1.56
Week 3	1.26	0.08	0.00	1.11	1.42
Week 4	1.34	0.08	0.00	1.19	1.50

Note: Regression results from the main specification using a Logit model. Number of person weeks = 4,907,584. We included controls for year of birth, calendar week, region of residence, country of birth, sex and parents' country of birth. Standard errors are clustered at the municipality level.

Figure 1: The development of delta and omicron cases in the estimated sample, week 48, 2021 – week 3, 2022. Share of sequenced samples with confirmed delta, omicron, and other results.
[Figure1.pdf]

Figure 2: Event-study analysis of pre- and post-SARS-COV-2 trends in odds ratios for GP utilization by variant
[Figure2.pdf]
Note: Estimates from logistic regression analysis on weekly data are reported. Adjusted odds ratios were estimated for indicator variables for relative week to omicron and delta and the omitted category defined as one week prior to infection, i.e week -1. Odds ratios were adjusted for age by including age indicators for 1 year age groups, sex, indicator for birth country and parental birth country, and municipality of residence. Standard errors are clustered at municipality level.

Confidential: For Review Only

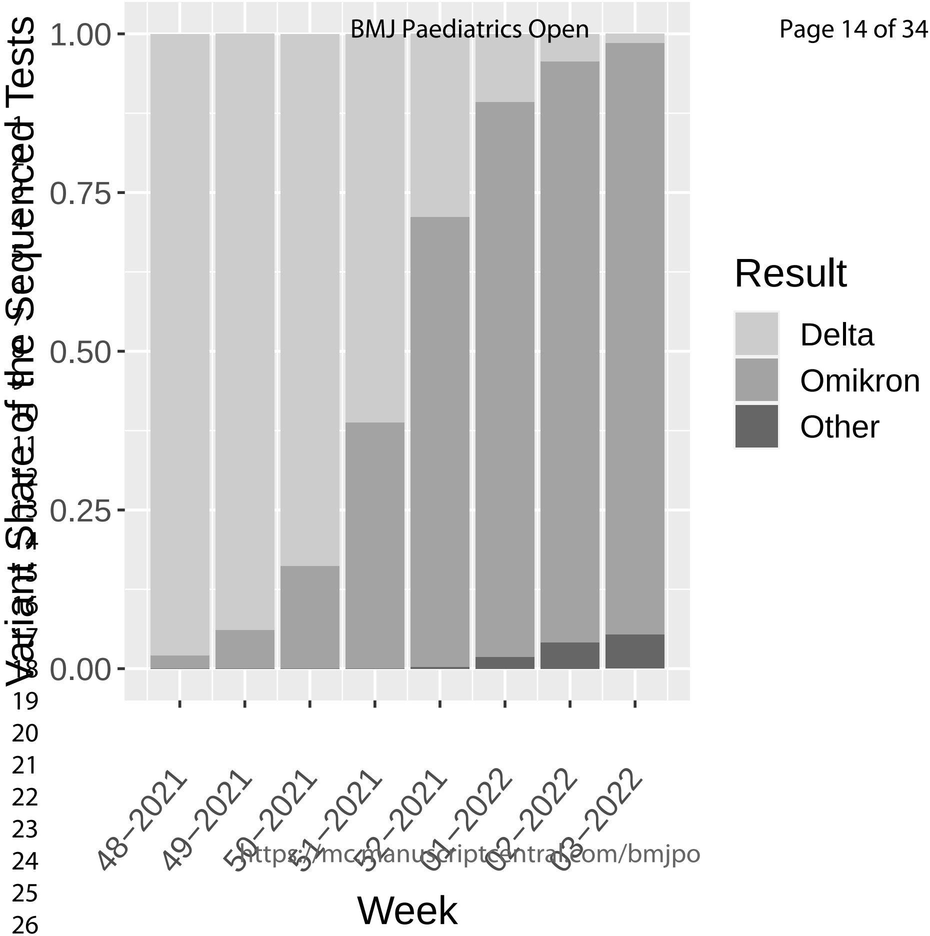
Variant Share of the Sequenced Tests
1.00
0.75
0.50
0.25
0.00
48−2021
49−2021
50−2021
51−2021
52−2021
01−2022
02−2022
03−2022
Week
Result
Delta
Omikron
Other

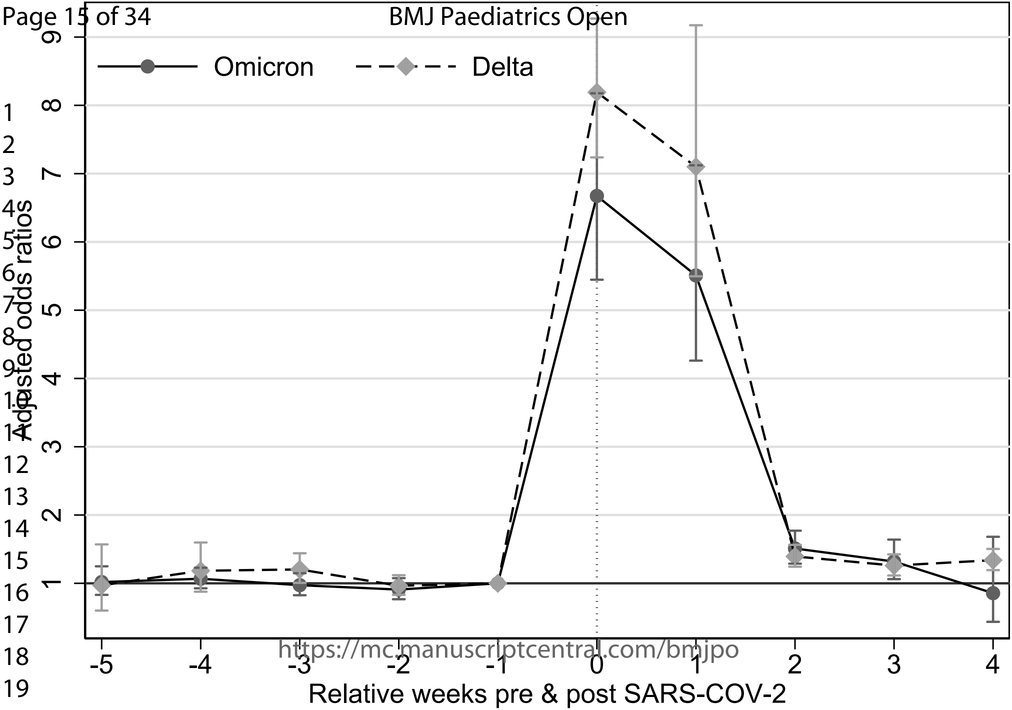

https://mc.manuscriptcentral.com/bmjpo

Supplementary online content

Arntzen et al. General practitioner visits after SARS-CoV-2 Omicron compared to the Delta variant in children: a prospective nationwide registry study

Confidential: For Review Only

Supplemental Methods

Part 1: Data sources

In this study, we utilized the Norwegian preparedness registry (Beredt C19) to retrieve and link nation-wide individual-level data originating from the sources listed in Supplementary Table 3.

Confidential: For Review Only

Table 1. Data sources in Beredt C19 used in this study and information obtained from each source.

**Name of data source**	**Information obtained**
The National Population Register	Resident of Norway November 1st 2021<br>Age<br>Sex<br>Municipality of residence<br>Country of birth<br>Parents' country of birth
The Norway Control and Payment of Health Reimbursement	All physical and electronic consultations with all general practitioners<br>Date of consultation
The Norwegian Surveillance System for Communicable Diseases	Date of sample of SARS-CoV-2 positive test<br><br>Classification of SARS-CoV-2 variants by targeted commercial or in-house PCR analyses for variant detection, Sanger sequencing of selected parts of the viral genome or whole genome sequencing.<br><br>For continuous surveillance purposes, 25% of SARS-CoV-2 positive samples or up to 100 samples per week per local laboratory is sent to a reference laboratory for whole genome sequencing. When Omicron emerged in Norway in late November 2021, the laboratories were requested to perform variant analyses locally on all positive samples. If this was not possible for capacity reasons, samples suspected to contain the Omicron variant were prioritized for variant analyses.
The National Immunization Register	Vaccination status by January 23rd, 2022<br><br>There is yet no recommended general vaccination for children below 12 years of age.

Information was linked at the individual level using the unique personal identification number (encrypted version) provided to every Norwegian resident at birth or upon immigration.

Confidential: For Review Only

Supplemental Table 2: Event study estimates from the sensitivity analysis of the effect of relative week according to week Delta and Omicron variant on GP use from the 15th of November 2021 until 6th of February 2022.

Relative Week	OR	Std. Err.	P-value	Lower CI	Upper CI
**Omicron**					
Week -5	1.21	0.06	0.00	1.09	1.33
Week -4	1.01	0.11	0.90	0.82	1.26
Week -3	1.10	0.08	0.23	0.94	1.28
Week -2	0.95	0.08	0.54	0.81	1.12
Week -1	0.95	0.07	0.48	0.81	1.10
Week 0	6.37	0.62	0.00	5.26	7.71
Week 1	5.18	0.77	0.00	3.86	6.94
Week 2	1.42	0.10	0.00	1.24	1.62
Week 3	1.47	0.11	0.00	1.27	1.70
Week 4	1.32	0.13	0.01	1.08	1.60
**Delta**					
Week -5	0.95	0.17	0.76	0.66	1.35
Week -4	0.93	0.09	0.45	0.77	1.12
Week -3	1.02	0.08	0.79	0.88	1.18
Week -2	1.00	0.06	0.98	0.90	1.12
Week -1	0.92	0.05	0.11	0.84	1.02
Week 0	8.19	0.54	0.00	7.20	9.32
Week 1	7.26	0.93	0.00	5.64	9.34
Week 2	1.35	0.08	0.00	1.20	1.52
Week 3	1.27	0.07	0.00	1.15	1.41
Week 4	1.33	0.07	0.00	1.20	1.48

Note: Regression results from the sensitivity analysis using a Logit model. Number of person weeks = 6,645,376. We included controls for year of birth, calendar week, region of residence, country of birth, sex and parents' country of birth. Standard errors are clustered at the municipality level.

Confidential: For Review Only

General practitioner visits after SARS-CoV-2 ~~Omicron~~omicron compared to the ~~Delta~~delta variant in children: a prospective nationwide registry study

Sigurd Storehaug Arntzen, MPhil[1], Hege Marie Gjefsen, PhD[1], Kjetil Elias Telle, PhD[1], Karin Magnusson, PhD[1], Ketil Størdal, MD PhD[2], Siri Håberg, MD PhD[3], Jonas Minet Kinge, PhD[134]

[1] Cluster for Health Services Research, Norwegian Institute of Public Health
[2] Division of Paediatric and Adolescent Medicine, University of Oslo
[3] Centre for Fertility and Health, Norwegian Institute of Public Health
[4] Department of Health Management and Health Economics, University of Oslo

Address correspondence to: Jonas Minet Kinge, Norwegian Institute of Public Health, Postboks 222, Skøyen, N-0213 Oslo
E-mail: jonas.minet.kinge@fhi.no.

Confidential: For Review Only

Conflict of Interest Disclosures (includes financial disclosures): The authors have no example conflicts of interest to disclose.

Funding/Support: Funding: The study was internally funded by the Norwegian Institute of Public Health and externally by the Research Council of Norway (project no 262700). The funding sources had no influence over the design and conduct of the study; collection, management, analysis, and interpretation of the data; preparation, review, or approval of the manuscript; or decision to submit the manuscript for publication.

~~**Word Count:** 508~~

SARS-CoV-2 in children leads to milder symptoms than in adults.[1] However, recent reports discuss whether the omicron variant might cause more severe symptoms than the delta variant in children.[2] In Norway, general practitioners (GP) serve as the first line health service and are free for children aged below 16. Based on national recommendations, healthy children below age 12 are mostly unvaccinated.[3] PCR tests are freely available and a proportion of these are screened for variants.

The aim of this analysis was to compare GP contacts among children in the five weeks after being infected with omicron or delta variants.

Using data from

Confidential: For Review Only

What is already known on this topic

SARS-CoV-2 in children is known to lead to an immediate increase in primary care utilization for 1-2 weeks after a positive test, before quickly falling back to pre-infection utilization levels. The difference in primary care utilization following infection with the delta and omicron virus variants is unknown.

What this study adds

A sudden increase in primary care use was observed in children who tested positive for SARS-CoV-2 in the two weeks after the test. After the first two weeks, the primary care utilization returned to pre-infection levels.

The increase in primary care utilization was higher for children infected with the delta than the omicron variant. Compared to the delta variant, the omicron variant is likely to result in less pressure on primary care services for infected children.

Confidential: For Review Only

Abstract:

Background: SARS-CoV-2 infection in children is followed by an immediate increase in primary care utilization. The difference in utilization following infection with the delta and omicron virus variants is unknown.

Objectives: To study whether general practitioner (GP) contacts are different in children infected with the omicron vs delta variant, for up to five weeks after the infection.

Setting: Primary care

Participants: All residents in Norway aged 0-10. After excluding 47,683 children with a positive test where the virus variant was not identified as delta or omicron and 474 children who were vaccinated, the primary study population consisted of 613,448 children.

Main outcome measures: GP visit

Methods: We estimated the difference in the weekly share visiting the GP after being infected with the delta or omicron variant to those in the study population who were either not tested or who tested negative, using an event-study design, controlling for calendar week of consultation, municipality fixed effects and sociodemographic factors in multivariate logistic regressions.

Results: Compared with pre-infection, increased GP utilization was found for children 1 and 2 weeks after testing positive for the omicron variant, with an odds ratio (OR) of 6.7 (Std.err: 0.69) in the first week and 5.5 (0.72) in the second. This increase was more pronounced for children with the delta variant, with an OR of 8.2 (0.52) in the first week and 7.1 (0.93) in the second. After 2 weeks, the GP utilization returned to pre-infection levels.

Conclusion: The omicron variant is likely to result in less pressure on primary health care services for children, compared with the delta variant.

Confidential: For Review Only

Introduction

SARS-CoV-2 for children is often referred to as leading to milder symptoms than in adults and recent studies found no increase in specialists care in children following infection.[1 2] However, recent reports discuss whether the omicron variant might cause more severe symptoms than the delta variant in children.[3 4]

Survey data can be used to determine patterns of health care need following infection, however, reporting and response bias may affect the accuracy of the estimates. Except for a few studies using registry data from a period dominated by delta and other earlier variants, little is known about the impact of SARS-CoV-2 on post-covid health care utilization in children[2]. Furthermore, we also do not know whether healthcare use among children and adolescents increases after initial omicron infection and whether this increase, if any, is comparable to the increase in utilization after infection with the delta variant. Such knowledge could be used to upscale or downscale the health care services.

Because the omicron variant has been found to cause less severe symptoms than the delta variant in adults, we hypotezized that a comparable pattern would be found for children. The aim of this analysis was to compare general practitioner (GP) contacts among children in the five weeks after being infected with the omicron or delta variants.

Methods

Data used for this project was from the Emergency Preparedness Register (Beredt C19). The establishment of an emergency preparedness register forms part of the legally mandated responsibilities of the Norwegian Institute of Public Health during epidemics. The Ethics Committee of South-East Norway confirmed (4 June 2020, #153204) that external ethical board review was not required.

Confidential: For Review Only

), we followed*Data Sources*

To estimate the share of children aged 0-10 years old visiting the GP after being infected with delta or omicron variant we used population wide longitudinal registry data from Norway. BeredtC19 is an emergency preparedness register that aims to rapidly provide ongoing overview and knowledge of the prevalence, causal relationships, and consequences of the COVID-19 epidemic in Norway. It includes information from various data sources that are updated daily, including the Norwegian surveillance system for communicable diseases (all testing and screening for SARS-CoV-2), the National Population Register (age, sex, country of birth, the National Immunization Register (vaccination status), and the Norway Control and Payment of Health Reimbursement (all physical and electronic consultations with all general practitioners). A more in-depth summary of the data sources used for our analysis are available in the Appendix Supplementary Table 1.

Study Population

We followed all Norwegian residents aged 0-10 years from the 29th of November 2021 until 23rd of January 2022 (Supplemental Table 1). The outcome variable was defined as at least one physical- or e-consultation with a GP in a week. Figure 1 shows the share of the sequenced PCR tests that were delta or omicron variant from the 29th of November until the 23rd of January. Children who tested positive but whose tests were not sequenced, and children who had been vaccinated, were excluded from the analysis.

The categorical outcome variable for GP contact was set to 1 if the individual had at least one physical- or e-consultation with a GP in a week, and 0 otherwise. In Norway, consultations with the GP are free for children aged below 16. The GPs serve as the first line in the health

Confidential: For Review Only

care services, prescribing medicines and performing simple procedures, and referring patients to further care when necessary.

Statistical design

Multivariate logistic regression was used to estimate adjusted odds ratios (aOR) with 95% confidence intervals (CI) for GP consultations. Exploiting the longitudinal nature of the data, we used an event-study design, controlling for calendar week of consultation, municipality fixed effects and sociodemographic controls.[5], controlling for calendar week of consultation, municipality fixed effects and sociodemographic characteristics. The temporal aORs of being infected by omicron and delta were estimated from 5 weeks before to 4 weeks after the week 0 of infection (Supplemental Methods part 2). The. We regress weeks to and from confirmed positive test on binary GP visits using:

$$y_{iw} = \theta_w + \theta_i + \sum_{k=-5,\ k\neq -1}^{k=5} \delta_{k(iw)}\alpha_k + \sum_{k=-5,\ k\neq -1}^{k=5} \sigma_{k(iw)}\beta_k + \epsilon_{iw}$$

Where y_{iw} is the outcome for individual i in week w. δ_k is a set of time variables, indicating that there are k weeks relative to the week in which the individual got infected with the delta virus. Similarly, σ_k indicates that there are k weeks relative to the week in which the individual got infected with the omicron virus. θ_w are event-specific fixed effects for calendar week w , θ_i denotes background characteristics for individual i including gender, age dummies, parental country background and the child's country background, as well as municipality fixed effects. ϵ_{iw} is the standard error clustered at the municipality level. Our primary parameters of interests are the α_k and β_k attached to the event time dummies. These will capture the changes in our outcome variable among the children infected with delta and omicron relative to the comparison group consisting of uninfected or non-tested children.

Confidential: For Review Only

The coefficients for k < 0 indicate the pre-trends prior to infection time, while k > 0 describe how the outcome changes after getting infected with either delta or omicron virus. Hence, the event-study allows for testing whether infected children followed the same patterns for GP visits as the noninfected~~.~~[4]. The reference category was 1 week prior to infection and noninfected individuals were included as controls. ~~We also varied the time period to explore the robustness of our findings.~~

~~In total, 661,587 children, aged 0–10 years, were included in the study. After excluding 47,683 children with positive test during the study period that were not sequenced and 474 children who were vaccinated, the primary study population consisted of 613,448 children comprising 4,907,584 person-weeks (Table). The delta variant was the dominant strain at the beginning and omicron was the dominant strain at the end of the study period (Supplemental Figure 1).~~

~~GP consultations in the weeks before infection were similar for the children with omicron and delta in the adjusted model (See the Figure and Supplemental Tables 2, 3 & 4). The aORs in the first and second week after positive tests were higher for delta than for omicron. Higher GP utilization was also found for children 4 and 5 weeks after testing positive for delta but returned to pre-infection levels in week 5 for omicron cases. Results from robustness control were similar (Supplemental Table 4).~~

~~Strengths of this study includes the use of data covering all Norwegian children and that both delta and omicron was circulating during our study period. A limitation of the study is that asymptomatic children may have been less likely to have had a test. If for example,~~

Confidential: For Review Only

~~asymptomatic SARS-CoV-2 was more common for the omicron virus, we might have underestimated the difference between the two strains.~~

In the Online Appendix we present robustness checks of our results by varying the time period of our analysis.

Patient and public involvement

No patients were involved in setting the research question or the outcome measures, nor were they involved in developing plans for recruitment, design, or implementation of the study. No patients were asked to advise on interpretation or writing up of results. There are no plans to disseminate the results of the research to study participants or the relevant patient community.

~~Funding~~

~~The study was internally funded by the Norwegian Institute of Public Health and externally by the Research Council of Norway (project no 262700).~~

~~Acknowledgments~~

~~The establishment of an emergency preparedness register forms part of the legally mandated responsibilities of the Norwegian Institute of Public Health during epidemics. The Ethics Committee of South-East Norway confirmed (4 June 2020, #153204) that external ethical board review was not required.~~

Results

In total, 661,587 children, aged 0–10 years, were residing in Norway in the study period. After excluding 474 children who were vaccinated and 47,665 children with positive tests

Confidential: For Review Only

during the study period where the virus variant was not identified as delta or omicron, the primary study population consisted of 613,448 children comprising 4,907,584 person-weeks.

Figure 1 shows that delta was the dominant virus variant at the beginning of our study period, while omicron was the dominant variant at the end of our study period. Persons with omicron were older and more often born abroad than persons with delta, though the sex distribution was similar (Table 1).

Table 2 shows descriptive statistics of the estimated sample, indicating that the fraction that visited the GP in the weeks before infection was similar for the children later infected with omicron and delta, and for those who either were not infected or not tested for SARS-CoV-2.

The event study plot showing higher GP utilization following omicron, compared with delta (Figure 2). The aORs of 8.19 (std.err: 0.52) in the first and 7.10 (0.93) in the second week after delta were higher than comparable estimates for omicron of 6.67 (0.69) and 5.51 (0.72) in week one and two respectively. Higher GP utilization was also found for children 4 weeks after testing positive for both omicron and delta. Higher utilization was also found five weeks after testing positive for delta but returned to pre-infection levels in week 5 for omicron cases (see Table 3 for details). Results from robustness control were similar (Supplemental Table 2).

Discussion

In this study of 613,448 Norwegian children aged 0-10 years, we found increased GP utilization for children 1 and 2 weeks after testing positive for the omicron variant, with similar and more pronounced increases for children with the delta variant. Our findings

Confidential: For Review Only

suggest that omicron will place less pressure on the primary care services per case. However, given the higher transmissibility of the omicron than the delta variant it can still lead to a high burden on the health care system.[6]

We could find no study for comparison of our findings, i.e. to our knowledge, the present study is the first to shed light on healthcare use following the omicron vs delta variant in children.

A number of reports of the omicron variant in adults suggests less serious illness[7], i.e. another possible interpretation of our findings may be that omicron is perceived as less severe, compared with the delta variant, lowering the parents' perceived need to seek healthcare after omicron than after delta. However, a key strength of our analysis is that both delta and omicron were circulating during our study period, and we adjusted for calendar week in our regression models. Hence, it is less likely that the results are driven by general changes in the inclination to contact the GP. In addition, it includes data covering all children residing in Norway, which reduce attrition and sample selection bias.

Based on national recommendations in Norway, healthy children below age 12 are mostly unvaccinated.[8] PCR tests are freely available and a proportion of these are screened for variants of concern. Several of the most populous municipalities in Norway had implemented mass testing in the schools before the first case of omicron was detected in Norway in late November 2021. However, throughout the study period, it was the norm that all positive rapid antigen home tests had to be confirmed with a PCR-test. The increasing use of rapid antigen home tests is therefore unlikely to seriously bias our estimates.

Confidential: For Review Only

A limitation of the study is that asymptomatic children might have been less likely to have a PCR-test. If, for example, asymptomatic SARS-CoV-2 infection was more common for the omicron than the delta variant, we might have underestimated the difference between them. Also, our findings may not be generalizable to countries without equal and free access to healthcare and PCR testing for SARS-CoV-2 for all inhabitants. Finally, for continuous surveillance purposes, 25% of SARS-CoV-2 positive samples or up to 100 samples per week per local laboratory is sent to a reference laboratory for whole genome sequencing. When omicron emerged in Norway in late November 2021, the laboratories were requested to perform variant analyses locally on all positive samples. If this was not possible for capacity reasons, samples suspected to contain the omicron variant was prioritized for variant analyses. This might have led to variation in the tests that were sequenced over time.

Conclusion

Our findings show that per positive test in children aged 0 to 10 years, the omicron variant is likely to result in less pressure on the health care system than the delta variant. However, the omicron variant is still associated with increased utilization, and it can still lead to a high burden on the health care system when the community omicron cases are high.

Confidential: For Review Only

References:

1. Lee B, Raszka WV, Jr. Dhochak N, Singhal T, Kabra S, et al. Pathophysiology of COVID-19 in Children: Looking Forward, Not Back.: why children fare better than adults? *The Indian Journal of Pediatrics* 2021;147(1). DOI: 10.1542/peds.2020-029736;87(7):537-46.

2. Magnusson K, Skyrud KD, Suren P, et al. Healthcare use in 700 000 children and adolescents for six months after covid-19: before and after register based cohort study. *BMJ* 2022;376:e066809. doi: 10.1136/bmj-2021-066809
3. Kozlov M. Does Omicron hit kids harder? Scientists are trying to find out.: Nature Web Site; 2022 [accessed 23.02. 2022.
3. 4. Sigal A, Milo R, Jassat W. Estimating disease severity of Omicron and Delta SARS-CoV-2 infections. *Nature Reviews Immunology* 2022 doi: 10.1038/s41577-022-00720-5
5. Bailey MJ, Goodman-Bacon A. The War on Poverty's Experiment in Public Medicine: Community Health Centers and the Mortality of Older Americans. *American Economic Review* 2015;105(3):1067-104. doi: 10.1257/aer.20120070
6. Jørgensen SB, Nygård K, Kacelnik O, et al. Secondary Attack Rates for Omicron and Delta Variants of SARS-CoV-2 in Norwegian Households. *JAMA* 2022 doi: 10.1001/jama.2022.3780
7. Veneti L, Bøås H, Kristoffersen AB, et al. Reduced risk of hospitalisation among reported COVID-19 cases infected with the SARS-CoV-2 Omicron BA. 1 variant compared with the Delta variant, Norway, December 2021 to January 2022. *Eurosurveillance* 2022;27(4):2200077.
8. Coronavirus vaccine - information for the public.: The Norwegian Institute of Public Health (NIPH).); 2022 [updated 28.02.2022 (. Available from: https://www.fhi.no/en/id/vaccines/coronavirus-immunisation-programme/coronavirus-vaccine/#vaccination-of-children-and-adolescents).
4. Dimick JB, Ryan AM. Methods for Evaluating Changes in Health Care Policy: The Difference-in-Differences Approach. JAMA 2014;312(22):2401-2402. DOI: 10.1001/jama.2014.16153 accessed 01.03. 2022.

Confidential: For Review Only

Table 1: Summary statistics of the estimation sample

	Omicron	Delta	Rest
**N**	7,046	14,369	~~613,448~~592,033
**Person-weeks**	56,368	114,952	4,~~907,584~~736,264
**Age, mean (SD)**	6.1 (3.1)	6.9 (2.8)	5.~~2~~1 (3.2)
**Born Foreign Country, (%)**	7.2	6.3	5.5
**Parents foreign Born Country, (%)**	53.7	43.0	38.8
**Boys, (%)**	51.3	51.4	51.3

Notes: Standard deviation in brackets

~~**Figure**~~

Table 2: Descriptive statistics of the estimated sample

	Delta			Omicron		
Relative week	Person-weeks with GP Appointment	Persons infected	Fraction with GP appointment	Person-weeks with GP appointment	Persons infected	Share with GP appointment
-5	19	692	0.03	145	4,975	0.03
-4	55	1,622	0.03	176	6,126	0.03
-3	113	3,452	0.03	147	6,600	0.02
-2	187	7,445	0.03	120	6,902	0.02
-1	259	11,636	0.02	125	7,024	0.02
0	2,285	14,369	0.16	846	7,046	0.12
1	1,795	14,351	0.13	674	6,263	0.11
2	335	14,177	0.02	136	4,133	0.03
3	282	13,677	0.02	60	2,071	0.03
4	304	12,747	0.02	17	920	0.02

Confidential: For Review Only

Table 3: Event study estimates of the effect of relative week according to week delta and omicron variant on GP use from the 29th of November 2021 until 23rd of January 2022.

Relative Week	OR	Std. Err.	P-value	Lower CI	Upper CI
**Omicron**					
Week -5	1.20	0.10	0.03	1.02	1.42
Week -4	1.02	0.11	0.86	0.83	1.25
Week -3	1.07	0.08	0.37	0.93	1.23
Week -2	0.97	0.08	0.75	0.83	1.15
Week -1	0.91	0.08	0.27	0.77	1.08
Week 0	6.67	0.69	0.00	5.45	8.18
Week 1	5.51	0.72	0.00	4.26	7.12
Week 2	1.51	0.12	0.00	1.29	1.77
Week 3	1.32	0.15	0.01	1.06	1.64
Week 4	0.86	0.29	0.65	0.44	1.68
**Delta**					
Week -5	0.58	0.37	0.39	0.16	2.04
Week -4	0.97	0.24	0.90	0.60	1.57
Week -3	1.18	0.18	0.27	0.88	1.60
Week -2	1.20	0.11	0.04	1.01	1.44
Week -1	0.96	0.07	0.63	0.83	1.12
Week 0	8.19	0.52	0.00	7.24	9.27
Week 1	7.10	0.93	0.00	5.50	9.17
Week 2	1.39	0.08	0.00	1.24	1.56
Week 3	1.26	0.08	0.00	1.11	1.42
Week 4	1.34	0.08	0.00	1.19	1.50

Note: Regression results from the main specification using a Logit model. Number of person weeks = 4,907,584. We included controls for year of birth, calendar week, region of residence, country of birth, sex and parents' country of birth. Standard errors are clustered at the municipality level.

Confidential: For Review Only

Figure 1: The development of delta and omicron cases in the estimated sample, week 48, 2021 – week 3, 2022. Share of sequenced samples with confirmed delta, omicron, and other results.
[Figure1.pdf]

Figure 2: Event-study analysis of pre- and post-SARS-COV-2 trends in odds ratios for GP utilization by variant
[~~Figure~~Figure2.pdf]
Note: Estimates from logistic regression analysis on weekly data are reported. Adjusted odds ratios were estimated for indicator variables for relative week to omicron and delta and the omitted category defined as one week prior to infection, i.e week -1. Odds ratios were adjusted for age by including age indicators for 1 year age groups, sex, indicator for birth country and parental birth country, and municipality of residence. Standard errors are clustered at municipality level.

Confidential: For Review Only

BMJ Paediatrics Open

General practitioner visits after SARS-CoV-2 omicron compared to the delta variant in children in Norway: a prospective nationwide registry study

Journal:	*BMJ Paediatrics Open*
Manuscript ID	bmjpo-2022-001502.R2
Article Type:	Original research
Date Submitted by the Author:	05-Jul-2022

Complete List of Authors:	Arntzen, Sigurd Storehaug; Norwegian Institute of Public Health<br>Gjefsen, Hege Marie; Norwegian Institute of Public Health<br>Telle, Kjetil Elias; Norwegian Institute of Public Health<br>Magnusson, Karin; Norwegian Institute of Public Health<br>Stordal, Ketil; Norwegian Institute of Public Health<br>Håberg, Siri; Norwegian Institute of Public Health, Institute Management and Staff<br>Kinge, Jonas; Norwegian Institute of Public Health

Keywords:	COVID-19

SCHOLARONE™
Manuscripts

I, the Submitting Author has the right to grant and does grant on behalf of all authors of the Work (as defined in the below author licence), an exclusive licence and/or a non-exclusive licence for contributions from authors who are: i) UK Crown employees; ii) where BMJ has agreed a CC-BY licence shall apply, and/or iii) in accordance with the terms applicable for US Federal Government officers or employees acting as part of their official duties; on a worldwide, perpetual, irrevocable, royalty-free basis to BMJ Publishing Group Ltd ("BMJ") its licensees and where the relevant Journal is co-owned by BMJ to the co-owners of the Journal, to publish the Work in this journal and any other BMJ products and to exploit all rights, as set out in our licence.

The Submitting Author accepts and understands that any supply made under these terms is made by BMJ to the Submitting Author unless you are acting as an employee on behalf of your employer or a postgraduate student of an affiliated institution which is paying any applicable article publishing charge ("APC") for Open Access articles. Where the Submitting Author wishes to make the Work available on an Open Access basis (and intends to pay the relevant APC), the terms of reuse of such Open Access shall be governed by a Creative Commons licence – details of these licences and which Creative Commons licence will apply to this Work are set out in our licence referred to above.

Other than as permitted in any relevant BMJ Author's Self Archiving Policies, I confirm this Work has not been accepted for publication elsewhere, is not being considered for publication elsewhere and does not duplicate material already published. I confirm all authors consent to publication of this Work and authorise the granting of this licence.

Confidential: For Review Only

General practitioner visits after SARS-CoV-2 omicron compared to the delta variant in children in Norway: a prospective nationwide registry study

Sigurd Storehaug Arntzen, MPhil[1], Hege Marie Gjefsen, PhD[1], Kjetil Elias Telle, PhD[1], Karin Magnusson, PhD[1], Ketil Størdal, MD PhD[2], Siri Håberg, MD PhD[3], Jonas Minet Kinge, PhD[134]

[1] Cluster for Health Services Research, Norwegian Institute of Public Health
[2] Division of Paediatric and Adolescent Medicine, University of Oslo
[3] Centre for Fertility and Health, Norwegian Institute of Public Health
[4] Department of Health Management and Health Economics, University of Oslo

Address correspondence to: Jonas Minet Kinge, Norwegian Institute of Public Health, Postboks 222, Skøyen, N-0213 Oslo
E-mail: jonas.minet.kinge@fhi.no.

Conflict of Interest Disclosures (includes financial disclosures): The authors have no example conflicts of interest to disclose.

Funding/Support: Funding: The study was internally funded by the Norwegian Institute of Public Health and externally by the Research Council of Norway (project no 262700). The funding sources had no influence over the design and conduct of the study; collection, management, analysis, and interpretation of the data; preparation, review, or approval of the manuscript; or decision to submit the manuscript for publication.

Confidential: For Review Only

Confidential: For Review Only

What is already known on this topic

SARS-CoV-2 in children is known to lead to an immediate increase in primary care utilization for 1-2 weeks after a positive test, before quickly falling back to pre-infection utilization levels. The difference in primary care utilization following infection with the delta and omicron virus variants is unknown.

What this study adds

A sudden increase in primary care use was observed in children who tested positive for SARS-CoV-2 in the two weeks after the test. After the first two weeks, the primary care utilization returned to pre-infection levels.

The increase in primary care utilization was higher for children infected with the delta than the omicron variant. Compared to the delta variant, the omicron variant is likely to result in less pressure on primary care services per infected child.

Abstract:

Background: SARS-CoV-2 infection in children is followed by an immediate increase in primary care utilization. The difference in utilization following infection with the delta and omicron virus variants is unknown.

Objectives: To study whether general practitioner (GP) contacts were different in children infected with the omicron vs delta variant, for up to four weeks after the week testing positive.

Setting: Primary care

Participants: All residents in Norway aged 0-10. After excluding 47,683 children with a positive test where the virus variant was not identified as delta or omicron and 474 children who were vaccinated, the primary study population consisted of 613,448 children.

Main outcome measures: GP visits

Methods: We estimated the difference in the weekly share visiting the GP after being infected with the delta or omicron variant to those in the study population who were either not tested or who tested negative, using an event-study design, controlling for calendar week of consultation, municipality fixed effects and sociodemographic factors in multivariate logistic regressions.

Results: Compared with pre-infection, increased GP utilization was found for children 1 and 2 weeks after testing positive for the omicron variant, with an odds ratio (OR) of 6.7 (Std.err: 0.69) in the first week and 5.5 (0.72) in the second. This increase was more pronounced for children with the delta variant, with an OR of 8.2 (0.52) in the first week and 7.1 (0.93) in the second. After 2 weeks, the GP utilization returned to pre-infection levels.

Conclusion: The omicron variant appears to have resulted in less primary health care interactions per infected child, compared with the delta variant.

Confidential: For Review Only

Introduction

SARS-CoV-2 for children is often referred to as leading to milder symptoms than in adults, and recent studies found no increase in specialists care in children following infection.[1 2] However, SARS-CoV-2 infection in children is followed by an immediate increase in primary care utilization, and recent reports have discussed whether the omicron variant might cause more severe symptoms than the delta variant in children.[2-4]

Survey data can be used to determine patterns of health care need following infection, however, reporting and response bias may affect the accuracy of the estimates. Except for a few studies using registry data from a period dominated by delta and other earlier variants, little is known about the impact of SARS-CoV-2 on post-covid health care utilization in children[2]. Furthermore, we also do not know whether healthcare use among children and adolescents increases after initial omicron infection and whether this increase, if any, is comparable to the increase in utilization after infection with the delta variant. Such knowledge could be used to upscale or downscale the health care services.

Because the omicron variant has been found to cause less severe symptoms than the delta variant in adults, we hypotesized that a comparable pattern would be found for children. The aim of this analysis was to compare general practitioner (GP) contacts among children in the four weeks after being infected with the omicron or delta variants.

Methods

Data used for this project was from the Emergency Preparedness Register (Beredt C19). The establishment of an emergency preparedness register forms part of the legally mandated responsibilities of the Norwegian Institute of Public Health during epidemics. The Ethics

Confidential: For Review Only

Committee of South-East Norway confirmed (4 June 2020, #153204) that external ethical board review was not required.

Data Sources

To estimate the share of children aged 0-10 years old visiting the GP after being infected with delta or omicron variant we used population wide longitudinal registry data from Norway. BeredtC19 is an emergency preparedness register that aims to rapidly provide ongoing overview and knowledge of the prevalence, causal relationships, and consequences of the COVID-19 epidemic in Norway. It includes information from various data sources that are updated daily, including the Norwegian surveillance system for communicable diseases (all testing and screening for SARS-CoV-2), the National Population Register (age, sex, country of birth, the National Immunization Register (vaccination status), and the Norway Control and Payment of Health Reimbursement (all physical and electronic consultations with all general practitioners). A more in-depth summary of the data sources used for our analysis is available in the Appendix Supplementary Table 1.

Study Population

We followed all Norwegian residents aged 0-10 years from the 29th of November 2021 until 23rd of January 2022. Figure 1 shows the share of the sequenced PCR tests that were delta or omicron variant from the 29th of November until the 23rd of January. Children who tested positive but whose tests were not sequenced, and children who had been vaccinated, were excluded from the analysis. The upper age cut-off at 10 was set as children who turned 11 at the start of the period, turned 12 during the study period and thus become eligible for the vaccine.

Confidential: For Review Only

The categorical outcome variable for GP contact was set to 1 if the individual had at least one physical- or e-consultation with a GP in a week, and 0 otherwise. In Norway, consultations with the GP are free for children aged below 16. The GPs serve as the first line in the health care services, prescribing medicines and performing simple procedures, and referring patients to further care when necessary.

Statistical design

We constructed a data set including one observation per individual per week from week 48 of 2021 until the 3rd week of 2022. Each week, each individuasl could either be registered with a GP consultation or not. For the individuals that were infected with covid-19, we constructed an index week of infection. For each index week, persons with omicron or delta were compared to persons without omicron or delta. Event time was indicated relative to the index week of covid-19 infection for each person and was our main variable of interest explaining primary health care use for omicron and delta variant, respectively.

Multivariate logistic regression was used to estimate adjusted odds ratios (aOR) with 95% confidence intervals (CI) for GP consultations. Exploiting the longitudinal nature of the data, we used an event-study design[5 6], controlling for calendar week of consultation, municipality fixed effects and sociodemographic characteristics.

The event-study is especially well suited when the timing of events varies across groups in the population, there is a high number of units not experiencing an event and any measured association might vary over time[7]. The approach is widely used in social sciences and now also increasingly popular in epidemiology and public health as it can display an abnormal shift in trend, and attribute that shift to an event[8-12].

https://mc.manuscriptcentral.com/bmjpo

Confidential: For Review Only

The temporal aORs of being infected by omicron and delta were estimated from 5 weeks before to 4 weeks after the week 0 of infection. We regress weeks to and from confirmed positive test on binary GP visits using the following expression:

$$\sigma(y_{iw}) = 1/(1 + e^{-y_{iw}})$$

$$y_{iw} = \theta_w + \theta_i + \sum_{k=-5,\ k\neq -1}^{k=5} \delta_{k(iw)}\alpha_k + \sum_{k=-5,\ k\neq -1}^{k=5} \sigma_{k(iw)}\beta_k$$

y_{iw} is the outcome for individual *i* in week *w,* i.e., GP visits. θ_w is a set of dummy variables for calendar week accounting for any changes in the inclination to visit a GP due to e.g., capacity constraints or holidays. θ_i denotes background characteristics for individual *i* including gender, age dummies, parental country background and the child's country background, as well as municipality fixed effects. δ_k is a set of time dummy variables, indicating the event time, i.e., the number of weeks *k* relative to the week in which the individual got infected with the delta virus, taking the value 0 if not being infected with the delta virus. Similarly, σ_k is a set of dummy variables for event time in the case of infection with the omicron variant. The week prior to the infection, k = -1, is used as our reference value, and this value is therefore omitted from the regression. Our primary parameters of interests were the β_k and α_k attached to the event time dummies. These captured the changes in the probability of visiting the GP among the children infected with delta and omicron relative to the comparison group consisting of uninfected or non-tested. ϵ_{iw} was the standard error clustered at the municipality level.

https://mc.manuscriptcentral.com/bmjpo

Confidential: For Review Only

The coefficients δ_k and σ_k for k < 0 indicated the GP use develops prior to infection time, while k > 0 described how the outcome changed after getting infected with either delta or omicron virus. Hence, the event-study framework allows for testing whether infected children followed the same patterns for GP visits as the noninfected prior to infection, and whether this pattern changed after the week 0 of infection. A discontinuous jump in the probability of visiting the GP around week 0 indicates an estimated difference in the probability of visiting the GP between the individuals infected with omicron or delta, and our comparison groups consisting of noninfected individuals.

In the Online Appendix we present robustness checks of our results by varying the time period of our analysis, age stratified analysis and results of analysis with additional adjustment for municipality-specific time trends.

Patient and public involvement

No patients were involved in setting the research question or the outcome measures, nor were they involved in developing plans for recruitment, design, or implementation of the study. No patients were asked to advise on interpretation or writing up of results. There are no plans to disseminate the results of the research to study participants or the relevant patient community.

Results

In total, 661,587 children, aged 0–10 years, were residing in Norway in the study period. After excluding 474 children who were vaccinated and 47,665 children with positive tests during the study period where the virus variant was not identified as delta or omicron, the primary study population consisted of 613,448 children comprising 4,907,584 person-weeks.

https://mc.manuscriptcentral.com/bmjpo

Confidential: For Review Only

Figure 1 shows that delta was the dominant virus variant at the beginning of our study period, while omicron was the dominant variant at the end of our study period. Persons with omicron were older and more often born abroad than persons with delta, though the sex distribution was similar (Table 1).

Table 2 shows descriptive statistics of the estimated sample, indicating that the fraction that visited the GP in the weeks before infection was similar for the children later infected with omicron and delta, and for those who either were not infected or not tested for SARS-CoV-2.

The event study plot shows higher GP utilization following omicron, compared with delta (Figure 2). The aORs of 8.19 (std.err: 0.52) in the first and 7.10 (0.93) in the second week after delta were higher than comparable estimates for omicron of 6.67 (0.69) and 5.51 (0.72) in week one and two respectively. Higher GP utilization was also found for children 4 weeks after testing positive for both omicron and delta. Higher utilization was also found four weeks after testing positive for delta but returned to pre-infection levels in week 4 for omicron cases (see Table 3 for details).

Results from when varying included time-period were similar (Supplemental Table 2).The results were also replicated in each age strata (Supplemental Tables 3A-C). The results were also robust to analysis including municipality-specific time trends (Supplemental Table 4).

Discussion

In this study of 613,448 Norwegian children aged 0-10 years, we found increased GP utilization for children 1 and 2 weeks after testing positive for the omicron variant, with

Confidential: For Review Only

similar and more pronounced increases for children with the delta variant. Our findings suggest that omicron will place less pressure on the primary care services per case.

In the week following positive test 16% of children with delta visited the GP, compared with 12% with omicron. After one week, this percent dropped to 13 and 11 percent for children with delta and omicron, respectively. This suggest less pressure on the services from omicron. However, if caseload is substantially increased, it might outweigh the reduced pressure per case. The overall pressure on the health care system is a product of how many are infected and their inclination to use the health care system. Even if the inclination is overall lower with omicron, the pressure on the health care system can be higher if the number of infected is sufficiently high.

A number of reports of the omicron variant in adults suggested less serious illness already a few weeks after the initial reports of the new variant[13]. Thus, our findings may be affected by parents' perceived less need to seek healthcare after omicron in their children, than after delta. However, a key strength of our analysis was that both delta and omicron were circulating during our study period, and the parents were not informed which strain they were infected with. Hence, given that we adjust for overall time trend, it is unlikely that a difference in the perceived relative severity of the two strains was the main driver of our results. One exception is that if infection with the delta and omicron variant differed in symptoms, known to the parents, the parents could have reacted accordingly.

There might very well have been fluctuations in the inclination for GP visits over time. We adjusted for calendar week in our regression models, making it unlikely that the results were driven by general changes in the inclination to contact the GP. As both virus strains were

https://mc.manuscriptcentral.com/bmjpo

Confidential: For Review Only

present in all weeks included in our analysis, changes due to e.g. high pressure on GPs or an overall impression of omicron being less severe should be addressed by the week-fixed effects. In addition, our analysis included data covering all children residing in Norway, which reduced attrition and sample selection bias.

Based on national recommendations in Norway, healthy children below age 12 are mostly unvaccinated.[14] PCR tests are freely available and a proportion of these are screened for variants of concern. Several of the most populous municipalities in Norway had implemented mass testing in the schools before the first case of omicron was detected in Norway in late November 2021. However, throughout the study period, it was the norm that all positive rapid antigen home tests had to be confirmed with a PCR-test. The increasing use of rapid antigen home tests was therefore unlikely to seriously bias our estimates.

A limitation of the study was that asymptomatic children might have been less likely to have a PCR-test. If, for example, asymptomatic SARS-CoV-2 infection was more common for the omicron than the delta variant, we might have underestimated the difference between them. Also, our findings may not be generalizable to countries without equal and free access to healthcare and PCR testing for SARS-CoV-2 for all inhabitants. Finally, there have been variation in the tests that were sequenced over time. For capacity reasons, samples suspected to contain the omicron variant were prioritized for variant analyses for a part of the period. This might have led to variation in the tests that were sequenced over time and therefore potentially the composition of the groups.

Conclusion

https://mc.manuscriptcentral.com/bmjpo

Confidential: For Review Only

Our findings showed that per positive test in children aged 0 to 10 years, the omicron variant was likely to result in fewer consultations per positive tested children, than the delta variant. However, the omicron variant was still associated with higher total number of consultations, and could lead to a high burden on the health care system when the number of children infected with omicron are high

References:

1. Dhochak N, Singhal T, Kabra S, et al. Pathophysiology of COVID-19: why children fare better than adults? *The Indian Journal of Pediatrics* 2020;87(7):537-46.
2. Magnusson K, Skyrud KD, Suren P, et al. Healthcare use in 700 000 children and adolescents for six months after covid-19: before and after register based cohort study. *BMJ* 2022;376:e066809. doi: 10.1136/bmj-2021-066809
3. Kozlov M. Does Omicron hit kids harder? Scientists are trying to find out: Nature Web Site; 2022 [accessed 23.02. 2022.
4. Sigal A, Milo R, Jassat W. Estimating disease severity of Omicron and Delta SARS-CoV-2 infections. *Nature Reviews Immunology* 2022 doi: 10.1038/s41577-022-00720-5
5. Sun L, Abraham S. Estimating dynamic treatment effects in event studies with heterogeneous treatment effects. *Journal of Econometrics* 2021;225(2):175-99. doi: https://doi.org/10.1016/j.jeconom.2020.09.006
6. Dimick JB, Ryan AM. Methods for Evaluating Changes in Health Care Policy: The Difference-in-Differences Approach. *JAMA* 2014;312(22):2401-02. doi: 10.1001/jama.2014.16153
7. Goodman-Bacon A. Difference-in-differences with variation in treatment timing. *Journal of Econometrics* 2021;225(2):254-77.
8. Ly DP, Essien UR, Olenski AR, et al. Affirmative action bans and enrollment of students from underrepresented racial and ethnic groups in US public medical schools. *Annals of Internal Medicine* 2022
9. Auger N, Naimi AI, Smargiassi A, et al. Extreme heat and risk of early delivery among preterm and term pregnancies. *Epidemiology* 2014:344-50.
10. Abouk R, Pacula RL, Powell D. Association between state laws facilitating pharmacy distribution of naloxone and risk of fatal overdose. *JAMA internal medicine* 2019;179(6):805-11.
11. Schnake-Mahl AS, O'Leary G, Mullachery PH, et al. The impact of keeping indoor dining closed on COVID-19 rates among large US cities: a quasi-experimental design. *Epidemiology* 2021;33(2):200-08.
12. Ghosh AK, Venkatraman S, Soroka O, et al. THE EFFECT OF SOCIAL DISTANCING MEASURES ON COVID-19 RELATED HOSPITALIZATION IN NEW YORK: AN EVENT STUDY EXAMINING THE ROLE OF OVERCROWDED HOUSLNG. *Journal of General Internal Medicine* 2021:S77-S77.

Confidential: For Review Only

13. Veneti L, Bøås H, Kristoffersen AB, et al. Reduced risk of hospitalisation among reported COVID-19 cases infected with the SARS-CoV-2 Omicron BA. 1 variant compared with the Delta variant, Norway, December 2021 to January 2022. *Eurosurveillance* 2022;27(4):2200077.
14. Coronavirus vaccine - information for the public: The Norwegian Institute of Public Health (NIPH); 2022 [updated 28.02.2022. Available from: https://www.fhi.no/en/id/vaccines/coronavirus-immunisation-programme/coronavirus-vaccine/#vaccination-of-children-and-adolescents accessed 01.03. 2022.

Table 1: Summary statistics of the estimation sample

	Omicron	Delta	Rest
**N**	7,046	14,369	592,033
**Person-weeks**	56,368	114,952	4,736,264
**Age, mean (SD)**	6.1 (3.1)	6.9 (2.8)	5.1 (3.2)
**Born Foreign Country, (%)**	7.2	6.3	5.5
**Parents foreign Born Country, (%)**	53.7	43.0	38.8
**Boys, (%)**	51.3	51.4	51.3

Notes: Standard deviation in brackets

Table 2: Descriptive statistics of the estimated sample

	Omicron			Delta		
Relative week	Person-weeks with GP appointment	Persons infected	Share with GP appointment	Person-weeks with GP Appointment	Persons infected	Fraction with GP appointment
-5	145	4,975	0.03	19	692	0.03
-4	176	6,126	0.03	55	1,622	0.03
-3	147	6,600	0.02	113	3,452	0.03
-2	120	6,902	0.02	187	7,445	0.03
-1	125	7,024	0.02	259	11,636	0.02
0	846	7,046	0.12	2,285	14,369	0.16
1	674	6,263	0.11	1,795	14,351	0.13
2	136	4,133	0.03	335	14,177	0.02
3	60	2,071	0.03	282	13,677	0.02
4	17	920	0.02	304	12,747	0.02

Confidential: For Review Only

Table 3: Event study estimates of the effect of relative week according to week delta and omicron variant on GP use from the 29th of November 2021 until 23rd of January 2022.

Relative Week	OR	Std. Err.	P-value	Lower CI	Upper CI	Test for equal OR (P-value)*
**Omicron**						
Week -5	1.02	0.11	0.86	0.83	1.25	0.85
Week -4	1.07	0.08	0.37	0.93	1.23	0.53
Week -3	0.97	0.08	0.75	0.83	1.15	0.13
Week -2	0.91	0.08	0.27	0.77	1.08	0.62
Week -1	1					
Week 0	6.67	0.69	0.00	5.45	8.18	0.01
Week 1	5.51	0.72	0.00	4.26	7.12	<0.01
Week 2	1.51	0.12	0.00	1.29	1.77	0.46
Week 3	1.32	0.15	0.01	1.06	1.64	0.70
Week 4	0.86	0.29	0.65	0.44	1.68	0.18
**Delta**						
Week -5	0.97	0.24	0.90	0.60	1.57	
Week -4	1.18	0.18	0.27	0.88	1.60	
Week -3	1.20	0.11	0.04	1.01	1.44	
Week -2	0.96	0.07	0.63	0.83	1.12	
Week -1	1				1	
Week 0	8.19	0.52	0.00	7.24	9.27	
Week 1	7.10	0.93	0.00	5.50	9.17	
Week 2	1.39	0.08	0.00	1.24	1.56	
Week 3	1.26	0.08	0.00	1.11	1.42	
Week 4	1.34	0.08	0.00	1.19	1.50	

Note: Regression results from the main specification using a Logit model. Number of person weeks = 4,906,952. We included controls for year of birth, calendar week, region of residence, country of birth, sex and parents' country of birth. Standard errors were clustered at the municipality level.
* the column shows p-values from Wald-tests of equal OR for omicron versus delta, based on the regression output.

Confidential: For Review Only

Figure 1: The development of delta and omicron cases in the estimated sample, week 48, 2021 – week 3, 2022. Share of sequenced samples with confirmed delta, omicron, and other results.
[Figure1.pdf]

Figure 2: Event-study analysis of pre- and post-SARS-COV-2 trends in odds ratios for GP utilization by variant
[Figure2.pdf]
Note: Estimates from logistic regression analysis on weekly data are reported. Adjusted odds ratios were estimated for indicator variables for relative week to omicron and delta and the omitted category defined as one week prior to infection, i.e week -1. Odds ratios were adjusted for age by including age indicators for 1 year age groups, sex, indicator for birth country and parental birth country, and municipality of residence. Standard errors were clustered at municipality level.

Confidential: For Review Only

BMJ Paediatrics Open

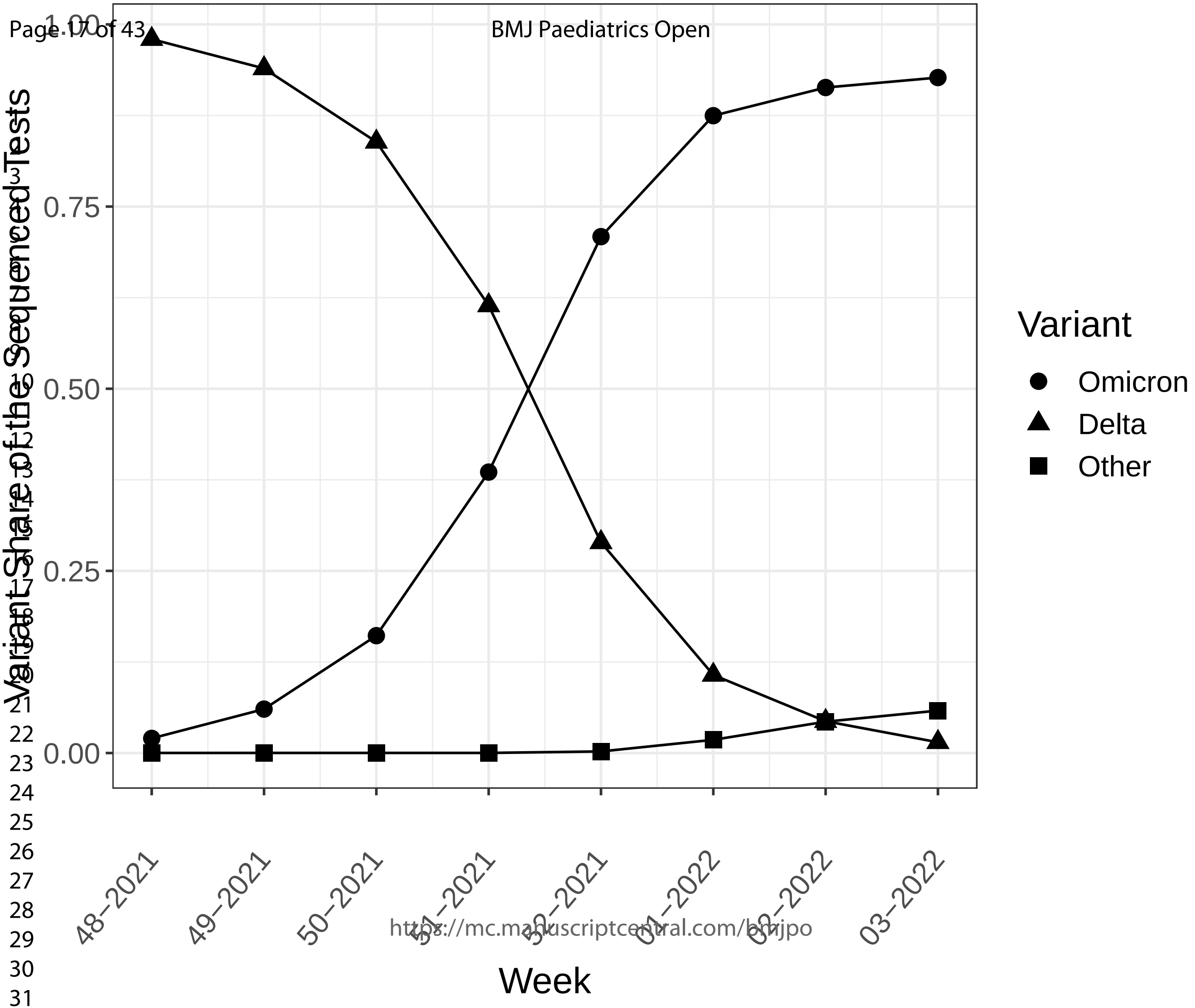

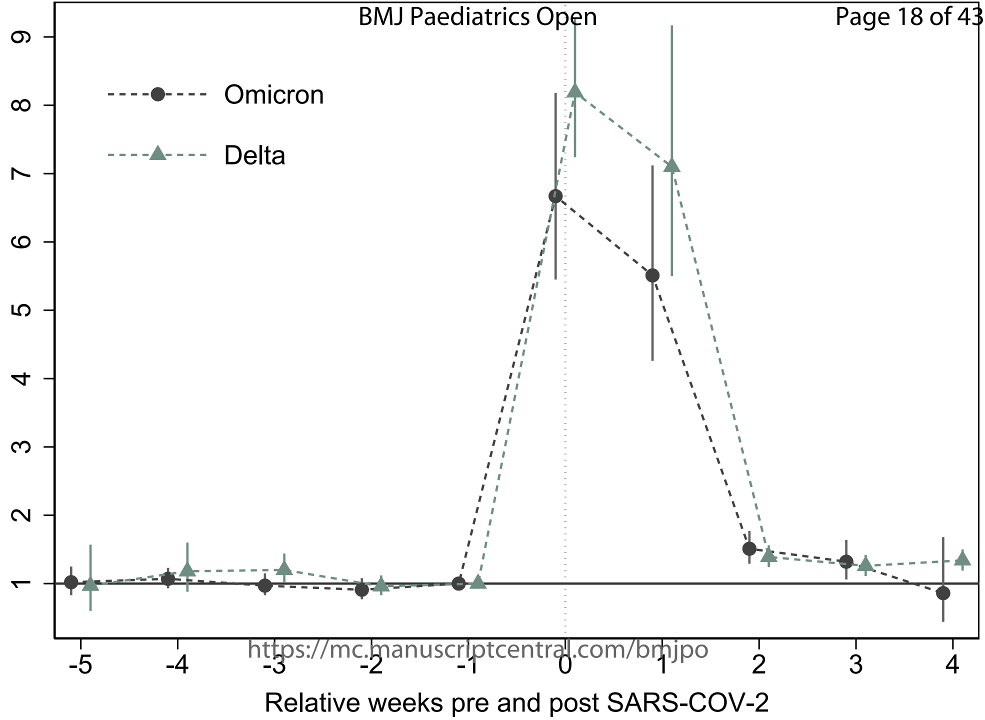

https://mc.manuscriptcentral.com/bmjpo

Supplementary online content

Arntzen et al. General practitioner visits after SARS-CoV-2 omicron compared to the delta variant in children in Norway: a prospective nationwide registry study

Confidential: For Review Only

Supplemental Methods

Part 1: Data sources

In this study, we utilized the Norwegian preparedness registry (Beredt C19) to retrieve and link nation-wide individual-level data originating from the sources listed in Supplementary Table 3.

Table 1. Data sources in Beredt C19 used in this study and information obtained from each source.

**Name of data source**	**Information obtained**
The National Population Register	Resident of Norway November 1st 2021<br>Age<br>Sex<br>Municipality of residence<br>Country of birth<br>Parents' country of birth
The Norway Control and Payment of Health Reimbursement	All physical and electronic consultations with all general practitioners<br>Date of consultation
The Norwegian Surveillance System for Communicable Diseases	Date of sample of SARS-CoV-2 positive test<br><br>Classification of SARS-CoV-2 variants by targeted commercial or in-house PCR analyses for variant detection, Sanger sequencing of selected parts of the viral genome or whole genome sequencing.<br><br>For continuous surveillance purposes, 25% of SARS-CoV-2 positive samples or up to 100 samples per week per local laboratory is sent to a reference laboratory for whole genome sequencing. When Omicron emerged in Norway in late November 2021, the laboratories were requested to perform variant analyses locally on all positive samples. If this was not possible for capacity reasons, samples suspected to contain the Omicron variant were prioritized for variant analyses.
The National Immunization Register	Vaccination status by January 23rd, 2022<br><br>There is yet no recommended general vaccination for children below 12 years of age.

Confidential: For Review Only

Information was linked at the individual level using the unique personal identification number (encrypted version) provided to every Norwegian resident at birth or upon immigration.

Confidential: For Review Only

Supplemental Table 2: Event study estimates from the sensitivity analysis of the effect of relative week according to week Delta and Omicron variant on GP use from the 15th of November 2021 until 6th of February 2022.

Relative Week	OR	Std. Err.	P-value	Lower CI	Upper CI
**Omicron**					
Week -5	1.01	0.11	0.90	0.82	1.26
Week -4	1.10	0.08	0.23	0.94	1.28
Week -3	0.95	0.08	0.54	0.81	1.12
Week -2	0.95	0.07	0.48	0.81	1.10
Week -1	1			1	1
Week 0	6.37	0.62	0.00	5.26	7.71
Week 1	5.18	0.77	0.00	3.86	6.94
Week 2	1.42	0.10	0.00	1.24	1.62
Week 3	1.47	0.11	0.00	1.27	1.70
Week 4	1.32	0.13	0.01	1.08	1.60
**Delta**					
Week -5	0.93	0.09	0.45	0.77	1.12
Week -4	1.02	0.08	0.79	0.88	1.18
Week -3	1.00	0.06	0.98	0.90	1.12
Week -2	0.92	0.05	0.11	0.84	1.02
Week -1	1			1	1
Week 0	8.19	0.54	0.00	7.20	9.32
Week 1	7.26	0.93	0.00	5.64	9.34
Week 2	1.35	0.08	0.00	1.20	1.52
Week 3	1.27	0.07	0.00	1.15	1.41
Week 4	1.33	0.07	0.00	1.20	1.48

Note: Regression results from the sensitivity analysis using a Logit model. Number of person weeks = 6,645,376. We included controls for year of birth, calendar week, region of residence, country of birth, sex and parents' country of birth. Standard errors are clustered at the municipality level.

Supplemental Table 3A: Event study estimates from the sensitivity analysis of the effect of relative week according to week Delta and Omicron variant on GP use in children aged 0-1 years

Age 0-1						
	Odds	Ratio	Std. Err.	z	[95% Conf. Interval]	
**Omicron**						
Week -5	1.59	0.32	2.30	0.02	1.07	2.37

Confidential: For Review Only

Week -4	1.31	0.18	1.98	0.05	1.00	1.72
Week -3	1.02	0.15	0.12	0.91	0.76	1.37
Week -2	1.11	0.16	0.72	0.47	0.83	1.49
Week -1	1.00					
Week 0	5.61	0.64	15.13	0.00	4.48	7.01
Week 1	4.24	0.55	11.21	0.00	3.30	5.46
Week 2	1.36	0.28	1.49	0.14	0.91	2.03
Week 3	0.99	0.24	-0.04	0.97	0.62	1.59
Week 4	1.51	1.11	0.56	0.57	0.36	6.36
**Delta**						
Week -5	0.37	0.37	-1.00	0.32	0.05	2.60
Week -4	1.07	0.28	0.24	0.81	0.63	1.80
Week -3	1.36	0.29	1.42	0.16	0.89	2.07
Week -2	1.27	0.21	1.46	0.14	0.92	1.76
Week -1	1.00					
Week 0	5.75	0.52	19.39	0.00	4.82	6.87
Week 1	4.47	0.96	7.01	0.00	2.94	6.80
Week 2	1.17	0.18	1.02	0.31	0.86	1.60
Week 3	1.27	0.17	1.79	0.07	0.98	1.66
Week 4	1.78	0.24	4.22	0.00	1.36	2.33

Supplemental Table 3B: Event study estimates from the sensitivity analysis of the effect of relative week according to week Delta and Omicron variant on GP use in children aged 2-5 years

Age 2-5						
	Odds	Ratio	Std. Err.	z	[95% Conf. Interval]	
**Omicron**						
Week -5	1.15	0.12	1.35	0.18	0.94	1.41
Week -4	1.15	0.12	1.32	0.19	0.93	1.42
Week -3	1.00	0.12	0.03	0.98	0.79	1.28

Week -2	0.97	0.15	-0.19	0.85	0.72	1.31
Week -1	1.00					
Week 0	6.04	0.70	15.40	0.00	4.80	7.59
Week 1	4.82	0.71	10.75	0.00	3.62	6.43
Week 2	1.47	0.21	2.70	0.01	1.11	1.95
Week 3	1.48	0.25	2.30	0.02	1.06	2.08
Week 4	1.08	0.57	0.15	0.88	0.38	3.04
**Delta**						
Week -5	1.62	0.44	1.77	0.08	0.95	2.75
Week -4	1.31	0.28	1.25	0.21	0.86	1.98
Week -3	1.27	0.23	1.37	0.17	0.90	1.80
Week -2	1.11	0.15	0.75	0.45	0.85	1.44
Week -1	1.00					
Week 0	6.70	0.55	22.97	0.00	5.70	7.88
Week 1	5.66	0.62	15.78	0.00	4.56	7.02
Week 2	1.69	0.16	5.55	0.00	1.41	2.04
Week 3	1.25	0.12	2.38	0.02	1.04	1.50
Week 4	1.48	0.15	3.89	0.00	1.21	1.80

Supplemental Table 3C: Event study estimates from the sensitivity analysis of the effect of relative week according to week Delta and Omicron variant on GP use in children aged 6-10 years

Age 6-10						
	Odds	Ratio	Std. Err.	z	[95% Conf. Interval]	
**Omicron**						
Week -5	0.74	0.09	-2.45	0.01	0.58	0.94
Week -4	0.92	0.08	-0.97	0.33	0.79	1.08
Week -3	0.96	0.12	-0.37	0.71	0.75	1.21
Week -2	0.79	0.10	-1.77	0.08	0.61	1.03
Week -1	1.00					

Week 0	7.10	0.82	17.00	0.00	5.66	8.89
Week 1	6.07	0.83	13.12	0.00	4.63	7.94
Week 2	1.53	0.15	4.33	0.00	1.26	1.85
Week 3	1.28	0.18	1.74	0.08	0.97	1.69
Week 4	0.51	0.18	-1.92	0.05	0.25	1.01
**Delta**						
Week -5	0.72	0.25	-0.93	0.35	0.37	1.43
Week -4	1.15	0.20	0.79	0.43	0.82	1.60
Week -3	1.14	0.17	0.89	0.38	0.85	1.53
Week -2	0.85	0.10	-1.45	0.15	0.68	1.06
Week -1	1.00					
Week 0	9.39	0.62	33.97	0.00	8.25	10.69
Week 1	8.36	1.16	15.29	0.00	6.36	10.97
Week 2	1.35	0.09	4.26	0.00	1.17	1.54
Week 3	1.25	0.09	2.98	0.00	1.08	1.45
Week 4	1.15	0.10	1.59	0.11	0.97	1.38

Supplemental table 4: Event study estimates of the effect of relative week according to week delta and omicron variant on GP use from the 29th of November 2021 until 23rd of January 2022, adjusted for municipality-specific time trends.

Supplemetal Table 4: Event study estimates from the sensitivity analysis of the effect of relative week according to week Delta and Omicron variant on GP use, adjusted for continuous time-by-municipality fixed effects

justert for kommune-tids-trender							
		Odds	Std. Err.	z	p	[95% Conf. Interval]	
	Omicron						
	-5	1.02	0.11	0.24	0.81	0.84	1.25

	-4	1.07	0.08	0.96	0.34	0.93	1.23
	-3	0.97	0.08	-0.30	0.77	0.83	1.15
	-2	0.91	0.08	-1.12	0.27	0.77	1.08
	-1	0.00					
	0	6.65	0.69	18.30	0.00	5.43	8.14
	1	5.48	0.72	12.92	0.00	4.23	7.09
	2	1.50	0.12	5.10	0.00	1.29	1.76
	3	1.31	0.14	2.49	0.01	1.06	1.63
	4	0.85	0.29	-0.47	0.64	0.43	1.67
	Delta						
	-5	0.98	0.24	-0.07	0.94	0.61	1.59
	-4	1.19	0.18	1.13	0.26	0.88	1.61
	-3	1.21	0.11	2.07	0.04	1.01	1.44
	-2	0.97	0.07	-0.45	0.65	0.83	1.12
	-1	0.00					
	0	8.21	0.53	32.80	0.00	7.24	9.31
	1	7.10	0.93	14.96	0.00	5.49	9.18
	2	1.39	0.08	5.65	0.00	1.24	1.56
	3	1.26	0.08	3.67	0.00	1.11	1.42
	4	1.34	0.08	4.87	0.00	1.19	1.50

w Only

General practitioner visits after SARS-CoV-2 omicron compared to the delta variant in children in Norway: a prospective nationwide registry study

Sigurd Storehaug Arntzen, MPhil[1], Hege Marie Gjefsen, PhD[1], Kjetil Elias Telle, PhD[1], Karin Magnusson, PhD[1], Ketil Størdal, MD PhD[2], Siri Håberg, MD PhD[3], Jonas Minet Kinge, PhD[134]

[1] Cluster for Health Services Research, Norwegian Institute of Public Health
[2] Division of Paediatric and Adolescent Medicine, University of Oslo
[3] Centre for Fertility and Health, Norwegian Institute of Public Health
[4] Department of Health Management and Health Economics, University of Oslo

Address correspondence to: Jonas Minet Kinge, Norwegian Institute of Public Health, Postboks 222, Skøyen, N-0213 Oslo
E-mail: jonas.minet.kinge@fhi.no.

Conflict of Interest Disclosures (includes financial disclosures): The authors have no example conflicts of interest to disclose.

Funding/Support: Funding: The study was internally funded by the Norwegian Institute of Public Health and externally by the Research Council of Norway (project no 262700). The funding sources had no influence over the design and conduct of the study; collection, management, analysis, and interpretation of the data; preparation, review, or approval of the manuscript; or decision to submit the manuscript for publication.

Confidential: For Review Only

What is already known on this topic

SARS-CoV-2 in children is known to lead to an immediate increase in primary care utilization for 1-2 weeks after a positive test, before quickly falling back to pre-infection utilization levels. The difference in primary care utilization following infection with the delta and omicron virus variants is unknown.

What this study adds

A sudden increase in primary care use was observed in children who tested positive for SARS-CoV-2 in the two weeks after the test. After the first two weeks, the primary care utilization returned to pre-infection levels.

The increase in primary care utilization was higher for children infected with the delta than the omicron variant. Compared to the delta variant, the omicron variant is likely to result in less pressure on primary care services ~~for~~per infected ~~children~~child.

Confidential: For Review Only

Confidential: For Review Only

Abstract:

Background: SARS-CoV-2 infection in children is followed by an immediate increase in primary care utilization. The difference in utilization following infection with the delta and omicron virus variants is unknown.

Objectives: To study whether general practitioner (GP) contacts ~~are~~were different in children infected with the omicron vs delta variant, for up to ~~five~~four weeks after the ~~infection~~week testing positive.

Setting: Primary care

Participants: All residents in Norway aged 0-10. After excluding 47,683 children with a positive test where the virus variant was not identified as delta or omicron and 474 children who were vaccinated, the primary study population consisted of 613,448 children.

Main outcome measures: GP ~~visit~~visits

Methods: We estimated the difference in the weekly share visiting the GP after being infected with the delta or omicron variant to those in the study population who were either not tested or who tested negative, using an event-study design, controlling for calendar week of consultation, municipality fixed effects and sociodemographic factors in multivariate logistic regressions.

Results: Compared with pre-infection, increased GP utilization was found for children 1 and 2 weeks after testing positive for the omicron variant, with an odds ratio (OR) of 6.7 (Std.err: 0.69) in the first week and 5.5 (0.72) in the second. This increase was more pronounced for children with the delta variant, with an OR of 8.2 (0.52) in the first week and 7.1 (0.93) in the second. After 2 weeks, the GP utilization returned to pre-infection levels.

Conclusion: The omicron variant ~~is likely~~appears to ~~result~~have resulted in less ~~pressure on~~ primary health care ~~services for children~~interactions per infected child, compared with the delta variant.

Confidential: For Review Only

Confidential: For Review Only

Introduction

SARS-CoV-2 for children is often referred to as leading to milder symptoms than in adults, and recent studies found no increase in specialists care in children following infection.[~~1-2~~][1 2] However, SARS-CoV-2 infection in children is followed by an immediate increase in primary care utilization, and recent reports ~~discuss~~have discussed whether the omicron variant might cause more severe symptoms than the delta variant in children.[~~3-4~~][2-4]

Survey data can be used to determine patterns of health care need following infection, however, reporting and response bias may affect the accuracy of the estimates. Except for a few studies using registry data from a period dominated by delta and other earlier variants, little is known about the impact of SARS-CoV-2 on post-covid health care utilization in children[2]. Furthermore, we also do not know whether healthcare use among children and adolescents increases after initial omicron infection and whether this increase, if any, is comparable to the increase in utilization after infection with the delta variant. Such knowledge could be used to upscale or downscale the health care services.

Because the omicron variant has been found to cause less severe symptoms than the delta variant in adults, we ~~hypotezized~~hypotesized that a comparable pattern would be found for children. The aim of this analysis was to compare general practitioner (GP) contacts among children in the ~~five~~four weeks after being infected with the omicron or delta variants.

Methods

Data used for this project was from the Emergency Preparedness Register (Beredt C19). The establishment of an emergency preparedness register forms part of the legally mandated responsibilities of the Norwegian Institute of Public Health during epidemics. The Ethics

Confidential: For Review Only

Committee of South-East Norway confirmed (4 June 2020, #153204) that external ethical board review was not required.

Data Sources

To estimate the share of children aged 0-10 years old visiting the GP after being infected with delta or omicron variant we used population wide longitudinal registry data from Norway. BeredtC19 is an emergency preparedness register that aims to rapidly provide ongoing overview and knowledge of the prevalence, causal relationships, and consequences of the COVID-19 epidemic in Norway. It includes information from various data sources that are updated daily, including the Norwegian surveillance system for communicable diseases (all testing and screening for SARS-CoV-2), the National Population Register (age, sex, country of birth, the National Immunization Register (vaccination status), and the Norway Control and Payment of Health Reimbursement (all physical and electronic consultations with all general practitioners). -A more in-depth summary of the data sources used for our analysis areis available in the Appendix Supplementary Table 1.

Study Population

We followed all Norwegian residents aged 0-10 years from the 29th of November 2021 until 23rd of January 2022. Figure 1 shows the share of the sequenced PCR tests that were delta or omicron variant from the 29th of November until the 23rd of January. Children who tested positive but whose tests were not sequenced, and children who had been vaccinated, were excluded from the analysis. The upper age cut-off at 10 was set as children who turned 11 at the start of the period, turned 12 during the study period and thus become eligible for the vaccine.

Confidential: For Review Only

The categorical outcome variable for GP contact was set to 1 if the individual had at least one physical- or e-consultation with a GP in a week, and 0 otherwise. In Norway, consultations with the GP are free for children aged below 16. The GPs serve as the first line in the health care services, prescribing medicines and performing simple procedures, and referring patients to further care when necessary.

Statistical design

We constructed a data set including one observation per individual per week from week 48 of 2021 until the 3rd week of 2022. Each week, each individuasl could either be registered with a GP consultation or not. For the individuals that were infected with covid-19, we constructed an index week of infection. For each index week, persons with omicron or delta were compared to persons without omicron or delta. Event time was indicated relative to the index week of covid-19 infection for each person and was our main variable of interest explaining primary health care use for omicron and delta variant, respectively.

Multivariate logistic regression was used to estimate adjusted odds ratios (aOR) with 95% confidence intervals (CI) for GP consultations. Exploiting the longitudinal nature of the data, we used an event-study design[55 6], controlling for calendar week of consultation, municipality fixed effects and sociodemographic characteristics.

The event-study is especially well suited when the timing of events varies across groups in the population, there is a high number of units not experiencing an event and any measured association might vary over time[7]. The approach is widely used in social sciences and now also increasingly popular in epidemiology and public health as it can display an abnormal shift in trend, and attribute that shift to an event[8-12].

Confidential: For Review Only

The temporal aORs of being infected by omicron and delta were estimated from 5 weeks before to 4 weeks after the week 0 of infection. We regress weeks to and from confirmed positive test on binary GP visits using the following expression:

$$y_{iw} = \theta_w + \theta_i + \sum_{k=-5,\ k\neq -1}^{k=5} \delta_{k(iw)}\alpha_k + \sum_{k=-5,\ k\neq -1}^{k=5} \sigma_{k(iw)}\beta_k +$$

$$\sigma(y_{iw}) = 1/(1 + e^{-y_{iw}})$$

$$y_{iw} = \theta_w + \theta_i + \sum_{k=-5,\ k\neq -1}^{k=5} \delta_{k(iw)}\alpha_k + \sum_{k=-5,\ k\neq -1}^{k=5} \sigma_{k(iw)}\beta_k$$

$$\epsilon_{iw}$$

Where y_{iw} is the outcome for individual i in week w. δ_k, i.e., GP visits. θ_w is a set of timedummy variables, indicating that there are k weeks relative to the week in which the individual got infected with the delta virus. Similarly, σ_k indicates that there are k weeks relative to the week in which the individual got infected with the omicron virus. θ_w are event-specific fixed effects for calendar week w ,accounting for any changes in the inclination to visit a GP due to e.g., capacity constraints or holidays. θ_i denotes background characteristics for individual i including gender, age dummies, parental country background and the child's country background, as well as municipality fixed effects. ϵ_{iw}. δ_k is the standard error clustered ata set of time dummy variables, indicating the event time, i.e., the number of weeks k relative to the week in which the individual got infected with the delta virus, taking the value 0 if not being infected with the delta virus. Similarly, σ_k municipality level.is a set of dummy variables for event time in the case of infection with the omicron variant. The week prior to the infection, k = -1, is used as our reference value, and this value is therefore omitted from the regression. Our primary parameters of interests arewere the β_k and α_k and

Confidential: For Review Only

β_k attached to the event time dummies. These will capturecaptured the changes in our outcome variablethe probability of visiting the GP among the children infected with delta and omicron relative to the comparison group consisting of uninfected or non-tested. ϵ_{iw} children. was the standard error clustered at the municipality level.

The coefficients δ_k and σ_k for k < 0 indicateindicated the pre-trendsGP use develops prior to infection time, while k > 0 describedescribed how the outcome changeschanged after getting infected with either delta or omicron virus. Hence, the event-study framework allows for testing whether infected children followed the same patterns for GP visits as the noninfected. The reference category was 1 week prior to infection and noninfected individuals were included as controls prior to infection, and whether this pattern changed after the week 0 of infection. A discontinuous jump in the probability of visiting the GP around week 0 indicates an estimated difference in the probability of visiting the GP between the individuals infected with omicron or delta, and our comparison groups consisting of noninfected individuals.

In the Online Appendix we present robustness checks of our results by varying the time period of our analysis., age stratified analysis and results of analysis with additional adjustment for municipality-specific time trends.

Patient and public involvement

No patients were involved in setting the research question or the outcome measures, nor were they involved in developing plans for recruitment, design, or implementation of the study. No patients were asked to advise on interpretation or writing up of results. There are no plans to disseminate the results of the research to study participants or the relevant patient community.

Confidential: For Review Only

Results

In total, 661,587 children, aged 0–10 years, were residing in Norway in the study period. After excluding 474 children who were vaccinated and 47,665 children with positive tests during the study period where the virus variant was not identified as delta or omicron, the primary study population consisted of 613,448 children comprising 4,907,584 person-weeks.

Figure 1 shows that delta was the dominant virus variant at the beginning of our study period, while omicron was the dominant variant at the end of our study period. Persons with omicron were older and more often born abroad than persons with delta, though the sex distribution was similar (Table 1).

Table 2 shows descriptive statistics of the estimated sample, indicating that the fraction that visited the GP in the weeks before infection was similar for the children later infected with omicron and delta, and for those who either were not infected or not tested for SARS-CoV-2.

The event study plot ~~showing~~shows higher GP utilization following omicron, compared with delta (Figure 2). The aORs of 8.19 (std.err: 0.52) in the first and 7.10 (0.93) in the second week after delta were higher than comparable estimates for omicron of 6.67 (0.69) and 5.51 (0.72) in week one and two respectively. Higher GP utilization was also found for children 4 weeks after testing positive for both omicron and delta. Higher utilization was also found ~~five~~four weeks after testing positive for delta but returned to pre-infection levels in week ~~5~~4 for omicron cases (see Table 3 for details).

Results from ~~robustness control~~when varying included time-period were similar (Supplemental Table 2).-The results were also replicated in each age strata (Supplemental

Confidential: For Review Only

Tables 3A-C). The results were also robust to analysis including municipality-specific time trends (Supplemental Table 4).

Discussion

In this study of 613,448 Norwegian children aged 0-10 years, we found increased GP utilization for children 1 and 2 weeks after testing positive for the omicron variant, with similar and more pronounced increases for children with the delta variant. Our findings suggest that omicron will place less pressure on the primary care services per case. However, given the higher transmissibility of the omicron than the delta variant it can still lead to a high burden on the health care system.[6]

We could find no study for comparison of our findings, i.e. to our knowledge, the present study is the first to shed light on healthcare use following the omicron vs delta variant in children.

In the week following positive test 16% of children with delta visited the GP, compared with 12% with omicron. After one week, this percent dropped to 13 and 11 percent for children with delta and omicron, respectively. This suggest less pressure on the services from omicron. However, if caseload is substantially increased, it might outweigh the reduced pressure per case. The overall pressure on the health care system is a product of how many are infected and their inclination to use the health care system. Even if the inclination is overall lower with omicron, the pressure on the health care system can be higher if the number of infected is sufficiently high.

Confidential: For Review Only

A number of reports of the omicron variant in adults ~~suggests~~suggested less serious illness[7]~~,~~ ~~i.e. another possible interpretation of~~ already a few weeks after the initial reports of the new variant[13]. Thus, our findings may be ~~that omicron is perceived as less severe, compared with the delta variant, lowering the~~ affected by parents' perceived less need to seek healthcare after omicron in their children, than after delta. However, a key strength of our analysis ~~is~~was that both delta and omicron were circulating during our study period, and ~~we~~the parents were not informed which strain they were infected with. Hence, given that we adjust for overall time trend, it is unlikely that a difference in the perceived relative severity of the two strains was the main driver of our results. One exception is that if infection with the delta and omicron variant differed in symptoms, known to the parents, the parents could have reacted accordingly.

There might very well have been fluctuations in the inclination for GP visits over time. We adjusted for calendar week in our regression models~~. Hence,~~, making it ~~is less likely~~unlikely that the results ~~are~~were driven by general changes in the inclination to contact the GP. As both virus strains were present in all weeks included in our analysis, changes due to e.g. high pressure on GPs or an overall impression of omicron being less severe should be addressed by the week-fixed effects. In addition, ~~it includes~~our analysis included data covering all children residing in Norway, which ~~reduce~~reduced attrition and sample selection bias.

Based on national recommendations in Norway, healthy children below age 12 are mostly unvaccinated.~~[8]~~[14] PCR tests are freely available and a proportion of these are screened for variants of concern. Several of the most populous municipalities in Norway had implemented mass testing in the schools before the first case of omicron was detected in Norway in late November 2021. However, throughout the study period, it was the norm that all positive

Confidential: For Review Only

rapid antigen home tests had to be confirmed with a PCR-test. The increasing use of rapid antigen home tests iswas therefore unlikely to seriously bias our estimates.

A limitation of the study iswas that asymptomatic children might have been less likely to have a PCR-test. If, for example, asymptomatic SARS-CoV-2 infection was more common for the omicron than the delta variant, we might have underestimated the difference between them. -Also, our findings may not be generalizable to countries without equal and free access to healthcare and PCR testing for SARS-CoV-2 for all inhabitants. Finally, for continuous surveillance purposes, 25% of SARS-CoV-2 positive samples or up to 100 samples per week per local laboratory is sent to a reference laboratory for whole genome sequencing. When omicron emerged in Norway in late November 2021, the laboratories were requested to perform variant analyses locally on all positive samples. If this was not possible forthere have been variation in the tests that were sequenced over time. For capacity reasons, samples suspected to contain the omicron variant waswere prioritized for variant analyses. for a part of the period. This might have led to variation in the tests that were sequenced over time and therefore potentially the composition of the groups.

Conclusion

Our findings showshowed that per positive test in children aged 0 to 10 years, the omicron variant iswas likely to result in less pressure on the health care systemfewer consultations per positive tested children, than the delta variant. However, the omicron variant iswas still associated with increased utilizationhigher total number of consultations, and it can stillcould lead to a high burden on the health care system when the communitynumber of children infected with omicron cases are high.

Confidential: For Review Only

References:

1. Dhochak N, Singhal T, Kabra S, et al. Pathophysiology of COVID-19: why children fare better than adults? *The Indian Journal of Pediatrics* 2020;87(7):537-46.
2. Magnusson K, Skyrud KD, Suren P, et al. Healthcare use in 700 000 children and adolescents for six months after covid-19: before and after register based cohort study. *BMJ* 2022;376:e066809. doi: 10.1136/bmj-2021-066809
3. Kozlov M. Does Omicron hit kids harder? Scientists are trying to find out: Nature Web Site; 2022 [accessed 23.02. 2022.
4. Sigal A, Milo R, Jassat W. Estimating disease severity of Omicron and Delta SARS-CoV-2 infections. *Nature Reviews Immunology* 2022 doi: 10.1038/s41577-022-00720-5
5. ~~Bailey MJ, Goodman-Bacon A. The War on Poverty's Experiment in Public Medicine: Community Health Centers and the Mortality of Older Americans. *American Economic Review* 2015;105(3):1067-104. doi: 10.1257/aer.20120070~~Sun L, Abraham S. Estimating dynamic treatment effects in event studies with heterogeneous treatment effects. *Journal of Econometrics* 2021;225(2):175-99. doi: https://doi.org/10.1016/j.jeconom.2020.09.006
6. ~~Jørgensen SB, Nygård K, Kacelnik O, et al. Secondary Attack Rates~~Dimick JB, Ryan AM. Methods for ~~Omicron and Delta Variants of SARS-CoV-2~~Evaluating Changes in ~~Norwegian Households.~~Health Care Policy: The Difference-in-Differences Approach. *JAMA* ~~2022~~2014;312(22):2401-02. doi: 10.1001/jama.~~2022.3780~~2014.16153

~~7.~~7. Goodman-Bacon A. Difference-in-differences with variation in treatment timing. *Journal of Econometrics* 2021;225(2):254-77.

8. Ly DP, Essien UR, Olenski AR, et al. Affirmative action bans and enrollment of students from underrepresented racial and ethnic groups in US public medical schools. *Annals of Internal Medicine* 2022

9. Auger N, Naimi AI, Smargiassi A, et al. Extreme heat and risk of early delivery among preterm and term pregnancies. *Epidemiology* 2014:344-50.

10. Abouk R, Pacula RL, Powell D. Association between state laws facilitating pharmacy distribution of naloxone and risk of fatal overdose. *JAMA internal medicine* 2019;179(6):805-11.

11. Schnake-Mahl AS, O'Leary G, Mullachery PH, et al. The impact of keeping indoor dining closed on COVID-19 rates among large US cities: a quasi-experimental design. *Epidemiology* 2021;33(2):200-08.

12. Ghosh AK, Venkatraman S, Soroka O, et al. THE EFFECT OF SOCIAL DISTANCING MEASURES ON COVID-19 RELATED HOSPITALIZATION IN NEW YORK: AN EVENT STUDY EXAMINING THE ROLE OF OVERCROWDED HOUSLNG. *Journal of General Internal Medicine* 2021:S77-S77.

13. Veneti L, Bøås H, Kristoffersen AB, et al. Reduced risk of hospitalisation among reported COVID-19 cases infected with the SARS-CoV-2 Omicron BA. 1 variant compared with the Delta variant, Norway, December 2021 to January 2022. *Eurosurveillance* 2022;27(4):2200077.

~~8~~14. Coronavirus vaccine - information for the public: The Norwegian Institute of Public Health (NIPH); 2022 [updated 28.02.2022. Available from: https://www.fhi.no/en/id/vaccines/coronavirus-immunisation-programme/coronavirus-vaccine/#vaccination-of-children-and-adolescents accessed 01.03. 2022.

Table 1: Summary statistics of the estimation sample

Confidential: For Review Only

	Omicron	Delta	Rest
**N**	7,046	14,369	592,033
**Person-weeks**	56,368	114,952	4,736,264
**Age, mean (SD)**	6.1 (3.1)	6.9 (2.8)	5.1 (3.2)
**Born Foreign Country, (%)**	7.2	6.3	5.5
**Parents foreign Born Country, (%)**	53.7	43.0	38.8
**Boys, (%)**	51.3	51.4	51.3

Notes: Standard deviation in brackets

Confidential: For Review Only

Table 2: Descriptive statistics of the estimated sample

	DeltaOmicron			OmicronDelta		
Relative week	Person-weeks with GP Appointmentappointment	Persons infected	Fraction Share with GP appointment	Person-weeks with GP appointmentAppointment	Persons infected	ShareFraction with GP appointment
-5	19145	6924,975	0.03	14519	4,975692	0.03
-4	55176	1,6226,126	0.03	17655	6,1261,622	0.03
-3	113147	3,4526,600	0.0302	147113	6,6003,452	0.0203
-2	187120	7,4456,902	0.0302	120187	6,9027,445	0.0203
-1	259125	11,6367,024	0.02	125259	7,02411,636	0.02
0	8462,285	7,04614,369	0.1612	2,285846	14,3697,046	0.1216
1	6741,795	6,26314,351	0.1311	1,795674	14,3516,263	0.1113
2	335136	14,1774,133	0.0203	136335	4,13314,177	0.0302
3	28260	13,6772,071	0.0203	60282	2,07113,677	0.0302
4	30417	12,747920	0.02	17304	92012,747	0.02

Table 3: Event study estimates of the effect of relative week according to week delta and omicron variant on GP use from the 29th of November 2021 until 23rd of January 2022.

Relative Week	OR	Std. Err.	P-value	Lower CI	Upper CI	Test for equal OR (P-value)*
**Omicron**						
Week -5	1.2002	0.1011	0.0386	1.020.83	1.4225	0.85
Week -4	1.0207	0.1108	0.8637	0.8393	1.2523	0.53
Week -3	1.070.97	0.08	0.3775	0.9383	1.2315	0.13
Week -2	0.9791	0.08	0.7527	0.8377	1.1508	0.62
Week -1	0.911	0.08	0.27	0.77	1.08	
Week 0	6.67	0.69	0.00	5.45	8.18	0.01
Week 1	5.51	0.72	0.00	4.26	7.12	<0.01
Week 2	1.51	0.12	0.00	1.29	1.77	0.46
Week 3	1.32	0.15	0.01	1.06	1.64	0.70
Week 4	0.86	0.29	0.65	0.44	1.68	0.18
**Delta**						
Week -5	0.5897	0.3724	0.3990	0.1660	1.572.04	
Week -4	0.971.18	0.2418	0.9027	0.88	01.60	1.57
Week -3	1.1820	0.1811	0.2704	1.01 0.88	1.6044	
Week -2	0.961.20	0.1107	0.0463	0.831.01	1.4412	
Week -1	10.96	0.07	0.63	0.83	1.12	
Week 0	8.19	0.52	0.00	7.24	9.27	
Week 1	7.10	0.93	0.00	5.50	9.17	
Week 2	1.39	0.08	0.00	1.24	1.56	
Week 3	1.26	0.08	0.00	1.11	1.42	
Week 4	1.34	0.08	0.00	1.19	1.50	

Note: Regression results from the main specification using a Logit model. Number of person weeks = 4,907,584906,952. We included controls for year of birth, calendar week, region of residence, country of birth, sex and parents' country of birth. Standard errors arewere clustered at the municipality level.
* the column shows p-values from Wald-tests of equal OR for omicron versus delta, based on the regression output.

Confidential: For Review Only

Figure 1: The development of delta and omicron cases in the estimated sample, week 48, 2021 – week 3, 2022. Share of sequenced samples with confirmed delta, omicron, and other results.
[Figure1.pdf]

Figure 2: Event-study analysis of pre- and post-SARS-COV-2 trends in odds ratios for GP utilization by variant

[Figure2.pdf]

Note: Estimates from logistic regression analysis on weekly data are reported. Adjusted odds ratios were estimated for indicator variables for relative week to omicron and delta and the omitted category defined as one week prior to infection, i.e week -1. Odds ratios were adjusted for age by including age indicators for 1 year age groups, sex, indicator for birth country and parental birth country, and municipality of residence. Standard errors ~~are~~were clustered at municipality level.

Confidential: For Review Only
